# CDK1 structures reveal conserved and unique features of the essential cell cycle CDK

**DOI:** 10.1038/ncomms7769

**Published:** 2015-04-13

**Authors:** Nicholas R. Brown, Svitlana Korolchuk, Mathew P. Martin, Will A. Stanley, Rouslan Moukhametzianov, Martin E. M. Noble, Jane A. Endicott

**Affiliations:** 1 Department of Biochemistry, University of Oxford, South Parks Road, Oxford, Oxfordshire OX1 3QU, UK; 2 Newcastle Cancer Centre, Northern Institute for Cancer Research, Newcastle University, Paul O’Gorman Building, Framlington Place, Newcastle Upon Tyne, Tyne and Wear NE2 4HH, UK

## Abstract

CDK1 is the only essential cell cycle CDK in human cells and is required for successful completion of M-phase. It is the founding member of the CDK family and is conserved across all eukaryotes. Here we report the crystal structures of complexes of CDK1–Cks1 and CDK1–cyclin B–Cks2. These structures confirm the conserved nature of the inactive monomeric CDK fold and its ability to be remodelled by cyclin binding. Relative to CDK2–cyclin A, CDK1–cyclin B is less thermally stable, has a smaller interfacial surface, is more susceptible to activation segment dephosphorylation and shows differences in the substrate sequence features that determine activity. Both CDK1 and CDK2 are potential cancer targets for which selective compounds are required. We also describe the first structure of CDK1 bound to a potent ATP-competitive inhibitor and identify aspects of CDK1 structure and plasticity that might be exploited to develop CDK1-selective inhibitors.

## Abstract (web summary)

Cyclin-dependent kinases are the principal drivers of cell cycle progression. Here the authors present several crystal structures of Cdk1 in complex with cyclin B and/or the assembly factors Cks1/2 and a small molecule inhibitor to reveal key features of this essential mitotic kinase.

## Graphical abstract

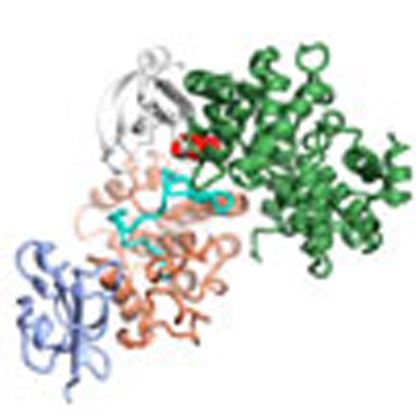

CDK1 is the founding member of the cyclin-dependent protein kinase (CDK) family and was first purified as a component of maturation promoting factor from unfertilized *Xenopus* eggs^1^. Subsequently human CDK1 was identified as a homologue of *Saccharomyces cerevisiae/Schizosaccharomyces pombe* Cdc28/Cdc2 (ref. 2) and cloned by complementation of *S. pombe Cdc2*^*+*^ (ref. 3; reviewed in ref. 4). CDK1 is partnered by cyclin A and cyclin B. Cyclin A is first expressed during late G1 where it initially binds to CDK2 and promotes S-phase (refs 5, 6; reviewed in ref. 7). Subsequently as cyclin A levels rise, it binds and rapidly activates CDK1 to generate detectable CDK1/cyclin A complexes by late S/G2. CDK1–cyclin A also regulates entry into mitosis and this complex persists until cyclin A destruction via the ubiquitin-proteasome system starting in early pro-metaphase^8^,^9^.

CDK1 is the only CDK to partner cyclin B, which starts to accumulate in S-phase^10^. Unlike CDK1–cyclin A whose activity tracks cyclin accumulation, the activity of CDK1–cyclin B is regulated by the opposing activities of Wee1 kinase and Cdc25 phosphatases, which together determine the extent of inhibitory phosphorylation within the CDK1 active site^11^ (reviewed in ref. 4). Activation of CDK1–cyclin B, first detected at centrosomes, signals the onset of mitosis, and CDK1 complexes of both cyclin A and B are required to ensure its successful completion^11^,^12^,^13^ (reviewed in refs 14, 15, 16). CDK1–cyclin B phosphorylates a large number of substrates^17^ and, in vertebrates, a number of phosphatases have been shown to counteract its activity^16^,^18^. Ultimately, however, CDK1 activity is depleted by the beginning of anaphase as a result of cyclin A and cyclin B ubiquitination and subsequent degradation through the respective activities of the APC/C E3 ubiquitin ligase and the proteasome^19^,^20^,^21^.

CDK1 is the only essential member of the CDK subfamily that drives cell cycle progression (reviewed in ref. 22). Deletion of the *CDK1* gene cannot be rescued by knock-in of its closest relative, *CDK2*, suggesting that in addition to its pattern and level of expression, CDK1 may possess unique structural features^23^. Cells that have been engineered to only express CDK1 can successfully replicate their DNA and divide, as under these circumstances CDK1 can form complexes with cyclins A, B, D and E^24^. *CDK1* conditional knockout mice are not viable, and the derived embryonic fibroblasts arrest in G2 subsequent to the induction of *CDK1* loss, frequently having undergone DNA re-replication as a result of elevated CDK2–cyclin A activity^25^. CDK1 has also been shown to have an essential role in meiosis where, in mouse oocytes, it is required for maturation^26^.

In addition to contributions from the cyclin subunit, the selection of substrates by CDKs can also be influenced by the presence of other accessory proteins. The first member of the Cks family was identified in *S. pombe* in a screen for genes that, in high copy number, can suppress the temperature-sensitive phenotype of certain *cdc2* alleles^27^. Biochemical studies in *Xenopus* suggested Cks proteins enhance the phosphorylation of selected CDK1 substrates at mitosis^28^ and recent work using *S. cerevisiae* as a model system has demonstrated their contribution to the recognition of CDK1 substrates primed by previous phosphorylation^29^,^30^. Cks1 binds to the CDK2 C-terminal domain in an orientation where it can be speculated that its phospho-amino-acid binding site would be positioned to enhance substrate binding within the CDK active site cleft^31^,^32^.

*A priori* the essential nature of CDK1 might be predicted to preclude its selection as a potential target for cancer treatment. However, with appropriate selection of molecular context, its unique ability to phosphorylate specific substrates may offer opportunities for therapeutic exploitation^33^,^34^,^35^,^36^. In this context, the validation of CDK1 as a clinical target has been hampered by the lack of potent and selective tool compounds. RO-3306 remains one of only a few inhibitors that show selectivity for CDK1 over other members of the CDK family^37^. Sequence differences between other members of the CDK family have permitted development of selective CDK4/6 and CDK9 inhibitors, a process that has been assisted by structural insights^38^,^39^,^40^,^41^.

In this paper we describe the crystal structure of monomeric CDK1 bound to Cks1 and the structures of a ternary CDK1–cyclin B–Cks2 complex in the apo form bound to a potent CDK1 inhibitor. These structures reveal the similarities and differences between an essential member of the CDK family and its close homologues.

## Results and Discussion

### Structure of CDK1–Cks1

**Figure 1:**
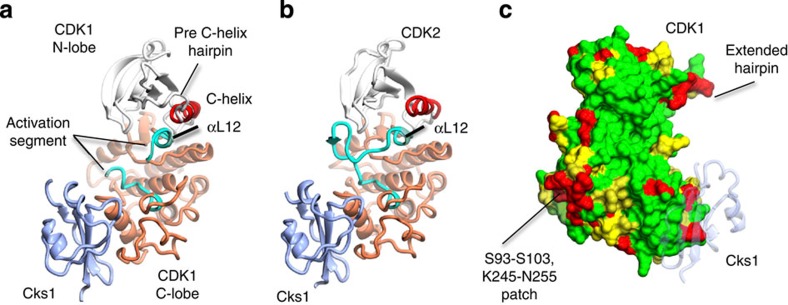
Structure of monomeric CDK1 bound to Cks1 and comparison with CDK2–Cks1. The complexes are shown in an identical view in panels **a** and **b**, and rotated by *circa* 60 degrees around a vertical axis in panel **c**. (**a**) CDK1–Cks1, (**b**) CDK2–Cks1 (PDB code 1BUH,^31^). In both panels the CDK N- and C-terminal lobes are coloured white and coral, respectively, and the Cks subunit is coloured ice-blue. Key regulatory elements are also highlighted: C-helix (CDK1 and CDK2 residues 47–57, red) and activation segment (CDK1 and CDK2 residues 146–173 and 145–172, respectively, cyan). (**c**) Sequence conservation between human CDK1 and CDK2 mapped onto the surface of CDK1 bound to Cks1. Identical residues (green), conserved residues (yellow) and non-conserved (red) as defined by CCP4MG^70^. The structure of Cks1 is shown in semi-transparent ribbon representation to indicate the location of its binding site.

The CDK1–Cks1 complex crystallizes with four CDK1–Cks1 heterodimers in the asymmetric unit to yield a lattice with large interfaces between the CDK1–Cks1 pairs (Fig. 1a, Supplementary Fig. 1 and Table 1). Monomeric CDK1 adopts a classical bi-lobal protein kinase fold that is in an inactive conformation. The C-helix of the N-terminal lobe is displaced out of the active site cleft and the non-phosphorylated activation segment (defined as the sequence between the conserved D146FG and SPE173 motifs) does not adopt a conformation capable of peptide substrate recognition. CDK1 and CDK2 share 65% sequence identity and the CDK1–Cks1 and CDK2–Cks1 structures (ref. 31, Fig. 1b) overlay well with an r.m.s.d. of 1.2 Å over 341 equivalent Cα atoms. Sequence identity between human CDKs 1 and 2 is relatively evenly distributed throughout the sequence, but breaks down for three stretches each of *circa* 10 residues that show a number of differences (Fig. 1c). CDK1 residues S93–S103, K245-N255 and the C-terminal tail starting at L290 are all found on the CDK1 surface. Notably the first two sequences are contiguous and encode, respectively, the loop linking αD and αE, and an extended peptide stretch that immediately precedes the start of αH. The first sequence encodes the only insertion between the human CDK1 and CDK2 sequences and the second lies beyond the residues that form part of the Cks1 binding site. The high degree of sequence conservation of these two contiguous sequences in CDK1 orthologues suggests that they may encode a potential site of CDK1–protein interaction.

The major structural differences between CDK2–Cks1 and CDK1–Cks1 occur in their respective activation segments, C-termini and loops that precede αC (Fig. 1). Both these and other minor differences, for example, between the Cks1 termini, are probably dictated in part by the close crystal packing (Supplementary Fig. 1). The CDK2 and CDK1 residues that form the Cks1 binding site are highly conserved and the relative disposition of the CDK C-terminal lobe and Cks1 subunit in each complex is highly conserved (Supplementary Fig. 1). The character of the CDK1 active site cleft beyond the ATP phosphate-binding site is shaped by residues 40–46 that precede the start of the C-helix and adopt an unusual extended hairpin (Fig. 1c). This structure originates in the peculiar geometries accessible to G43 and P45, and its conformation is probably dictated by lattice contacts. The sequence is disordered in the CDK2–Cks1 structure and adopts a more compact fold that contributes to the cyclin B interface in the CDK1–cyclin B–Cks2 structure.

The activation segment in monomeric CDK2 is relatively mobile but, in most monomeric CDK2 crystal structures, it forms a β-hairpin that contacts the glycine-rich loop and occludes the active site from solvent^42^. Like CDK2, the CDK1 glycine-rich loop is mobile, while residues 156–162, which form the core of the activation segment, do not have supporting electron density in any CDK1 molecule. At the start of the activation segment, the Cα positions of the DFG motif align closely with those of CDK2 and both kinases then adopt a short α-helical structure (αL12, Fig. 1a,b) that displaces the C-helix from the active site. The only difference between CDK1 and CDK2 in this region is the location of the DFG phenylalanine sidechain: in the CDK1 structure F147 is rotated back towards the carbonyl moiety of V64, whereas in CDK2 it points into the active site towards D185. At the other end of the activation segment, there are no notable differences between the CDK2 and CDK1 structures starting from CDK1 T166 when the CDK1 electron density map is once more interpretable. Beyond the activation segment the CDK1 C-terminal lobe is well-ordered adopting an all α-helical structure to which Cks1 binds. However, starting at L290, the CDK1 sequence is disordered in all four ncs-related CDK1 molecules. This C-terminal flexibility distinguishes the CDK1 structure from CDK2 where the equivalent residues wend along the back of the fold towards the hinge^42^,^43^.

### Structure of CDK1–cyclin B–Cks2

**Figure 2:**
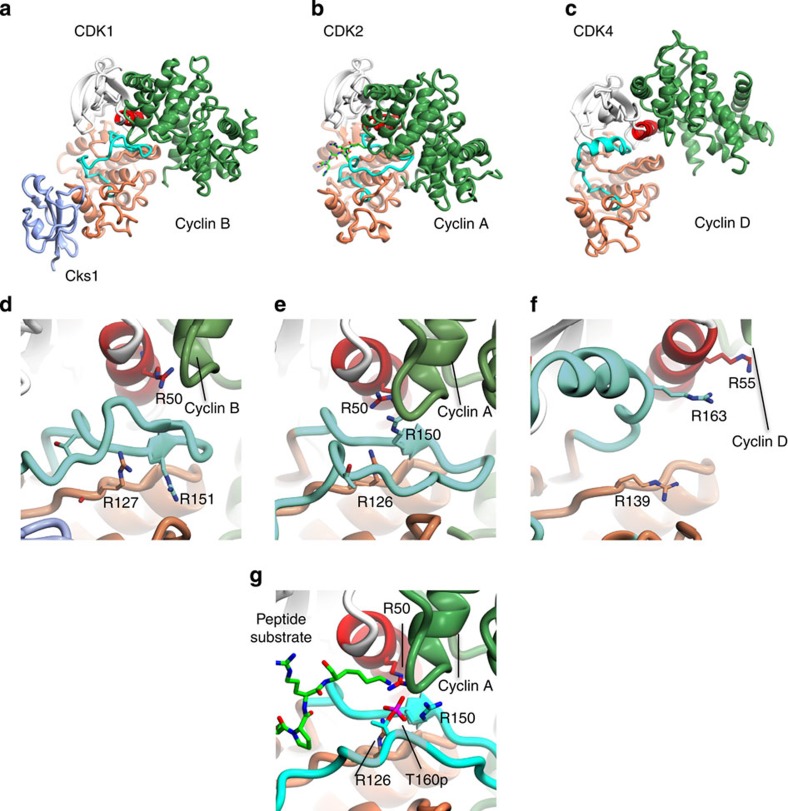
Structure of CDK1–cyclin B–Cks2 and a comparison with unphosphorylated CDK2–cyclin A and unphosphorylated CDK4–cyclin D3. (**a**,**d**) CDK1–cyclin B-Cks2, (**b**,**e**) CDK2–cyclin A (PDB code 1FIN,^43^), (**c**,**f**) CDK4–cyclin D3 (PDB code 3G33,^44^). Each complex in each row is shown in the same view. CDK structural elements and N- and C-terminal lobes are coloured according to the same scheme as used in Fig. 1, the cyclin subunits are coloured lawn green. A peptide substrate from PDB file 1QMZ is included in panel (**b**) to indicate the site of peptide substrate binding. The residues that compose the charge cluster surrounding CDK2 T160 are drawn in cylinder mode in (**e**). The equivalent residues of CDK1 (**d**) and CDK4 (**f**) are also shown. The fully composed charge cluster surrounding phosphorylated CDK2 T160 (PDB code 1QMZ,^50^) is shown in (**g**).

The structure of CDK1–cyclin B–Cks2 was determined at 2.8 Å resolution from a complex assembled from the individually purified proteins subsequent to recombinant protein expression in either insect (CDK1) or *E. coli* (cyclin B and Cks2) cells (Table 1). Overall the relative orientation of the CDK1 and cyclin B subunits is distinctive (Fig. 2a) and differs from structures previously reported for CDK2–cyclin A (ref. 43, Fig. 2b) and CDK4–cyclin D3 (ref. 44, Fig. 2c). An opening of the interface coupled with a twist between the two proteins relative to CDK2–cyclin A results in a re-orientation of the C-helix and fewer interactions between the cyclin B and CDK1 C-terminal domains. Overall the CDK–cyclin interface is nearly 30% smaller in CDK1–cyclin B (1,217 Å^2^ calculated using the CDK1–cyclin B–Cks1-Compound 23 structure, see below) than in CDK2–cyclin A (PDB code 1FIN, 1,697 Å^2^). The structures of monomeric and CDK1-bound cyclin B superimpose well (r.m.s.d. 0.92 Å over 252 equivalent Cα,^45^). The only additional significant feature in the CDK1–cyclin B structure is a helical ordering of the cyclin B C-terminal tail starting at P416.

In contrast to its disordered state in the CDK1/Cks1 structure, the CDK1 C-terminal tail forms an amphipathic helix (Supplementary Fig. 2a,c) which, by virtue of the crystal packing, binds to a symmetry-related cyclin B molecule at a site previously shown in cyclin A to bind to the CDK inhibitor p27^Kip1^ (ref. 46, Supplementary Fig. 2b,d). Although a crystallographic artefact, this lattice contact does suggest both a propensity of the C-terminal sequence of CDK1 to unfold away from the C-terminal CDK1 fold and for the complementary site on the cyclin B subunit to mediate diverse protein–protein interactions.

In the course of the normal cell cycle, CDK1 is found in complexes containing cyclin A and cyclin B, while CDK2 is found in complexes that contain cyclin A and cyclin E. To investigate the basis for this selectivity, we identified the sequence loci of cyclin B that mediate the relatively small CDK1/cyclin B interface, and compared their amino acid identity with that of the equivalent loci in cyclin A and cyclin E. In total, 18 residues of cyclin B lie within 3.6 Å of a residue of cyclin A (Supplementary Fig. 3). Of these, 10 are identical with equivalent amino acids of cyclin A, while only five are identical with the equivalent amino acids of cyclin E. In particular, residues Y170, Y177 and Y258 of cyclin B are conserved as aromatics in cyclin A and present large side chains that pack against the surface of CDK1, while cyclin E presents smaller amino acids at these positions (N112, I119 and L202). These observations suggest an explanation for why CDK1 binds preferentially to cyclins B and A rather than to cyclin E. By contrast, CDK2 forms a larger interface with its cognate cyclins. In the context of this larger interface, the sub-optimal interactions of the smaller cyclin E side chains may be better tolerated.

The CDK2–cyclin A and CDK4–cyclin D3 structures revealed differing responses of the CDK subunit to cyclin binding (Fig. 2b,c). Binding of cyclin A to CDK2 leads to melting of the CDK2 αL12 helix that, together with the concomitant movement of the C-helix, organizes key catalytic residues within the active site^43^. Through interactions with the cyclin A C-terminal cyclin box fold, the CDK2 activation segment adopts a conformation that requires little re-arrangement on T160 phosphorylation to recognize a peptide substrate. These changes also help to re-organize R50, R126 and R150 from the C-helix, catalytic loop and activation segment, respectively, so that they are poised to accept this conserved phosphothreonine residue (compare Fig. 2e,g).

In contrast, binding of cyclin D3 to CDK4 is not sufficient to drive CDK4 towards an active conformation (ref. 44, Fig. 2c). The CDK4 C-helix remains displaced from the active site and the activation segment, though structured, makes no contacts with the cyclin subunit and is not compatible with substrate binding. As a result R55, R139 and R163, the potential phosphorylated T172 ligands, are dispersed (Fig. 2f). Even when phosphorylated and cyclin bound, the CDK4–cyclin D1 active site remains in a conformation that appears unable to support catalysis suggesting a substrate-assisted catalytic mechanism^47^.

The CDK1–cyclin B structure is different again and, though closer in overall structure to CDK2–cyclin A than CDK4–cyclin D, it has features that distinguish it from its closest homologues (Fig. 2a, Supplementary Fig. 4). The activation segment of CDK1 does not extend across to make extensive contacts with the cyclin subunit as it does in CDK2 binary complexes (compare Fig. 2d,e). Instead, the sequence forms a short β-hairpin with residues F153 and G154 at the tip. Significant interactions within this region are made by F153, which sits within a cyclin B hydrophobic groove (Supplementary Fig. 4). As the CDK1 sequence heads back, cyclin B residues at the end of helix α3 loop down to occlude CDK1 I155 from solvent. The pruning back of the CDK1 activation segment creates a solvent-filled cleft between the CDK1 and cyclin B subunits completed at the back by the cyclin B N-terminal helix (Fig. 2a). This cleft is particularly marked in the structures of CDK4–cyclin D and CDK9–cyclin T^48^ and is not a feature of CDK2 bound to cyclin B^49^, suggesting it is a peculiarity of the CDK1 structure.

As a result of its curtailed excursion, the CDK1 activation segment puckers up at I157 so that the isoleucine side chain points towards the C-helix below the R50-E51 peptide bond (Supplementary Fig. 4). To accommodate this bulk, the C-helix and start of the activation segment at the DFG motif are displaced away from their positions in CDK2, respectively. further out and deeper into the active site cleft. As a consequence, the distance between the side chain carboxylate of E51 and the K33 episilon amino group is *circa* 5.0 Å compared with 3.1 Å in CDK2–cyclin A^43^. R50 is also moved with respect to its position in CDK2–cyclin A to form a hydrogen bond with the backbone carbonyl of cyclin B K257 (Supplementary Fig. 4). The two other predicted ligands of phosphoT161 (residues R127 and R151 of CDK1) are also displaced and engaged in a network of interactions centred around CDK1 Y181. CDK1 E163, the residue equivalent to CDK2 E162 that mimics phosphoT160 in the non-phosphorylated CDK2–cyclin A structure, is in a part of the CDK1 structure between H162 and V165 that cannot be built.

Despite ATP analogues being present in crystallization trials, there was no electron density to support ligand binding in the CDK1 active site. We hypothesize that this absence results from the relative orientation of the CDK1 N- and C-terminal lobes and the disposition of the K33-E51-D146 triad negatively impacting the binding of the ATP adenine ring and phosphate moieties, respectively. However, as we describe below, the CDK1 structure is flexible and binding of a potent ATP-competitive inhibitor induces an active site conformation that is more compatible with catalysis.

**Figure 3:**
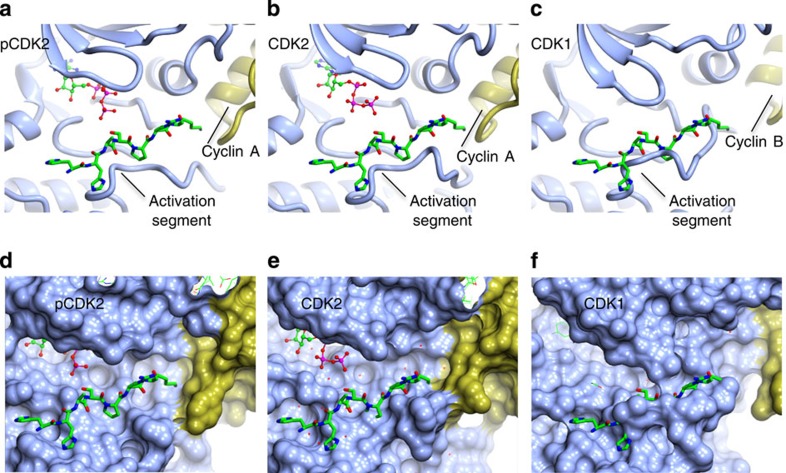
The CDK substrate binding site. (**a**,**d**) T160-phosphorylated CDK2–cyclin A (1QMZ,^50^); (**b**,**e**) unphosphorylated CDK2–cyclin A (PDB code 1FIN,^43^); (**c**,**f**) unphosphorylated CDK1–cyclin B. Each complex in each row is shown in the same view, pCDK and CDK designate the phosphorylated and unphosphorylated proteins, respectively. CDK and cyclin subunits are coloured ice-blue and gold, respectively. In all panels the peptide from 1QMZ is shown as a reference for the site of peptide substrate binding and is drawn in cylinder mode with carbon atoms coloured green. (**a**–**c**) Ribbon representation of the proteins. (**d–f**) Molecular surfaces of the proteins to highlight shape complementarity and/or steric clashes.

The activation segment forms a platform that recognizes the CDK substrate residues to either side of the site of phospho-transfer^50^. The interaction is mediated by β-sheet-style hydrogen bonds between the substrate and activation segment backbone carbonyl and amide groups augmented by complementary CDK pockets that recognize the side chains of residues at the P-3 to P+3 positions. Cyclin A binding to CDK2 remodels the CDK2 activation segment to a conformation in which the peptide substrate binding site is largely formed and requires only minor re-arrangement to adopt its fully active structure (Compare Fig. 3 panels a,d and b,e). However, the CDK1 activation segment makes fewer interactions with cyclin B, includes a disordered region and requires considerable re-arrangement to be able to recognize a peptide substrate (Fig. 3c,f).

These conformational differences between the CDK1 and CDK2 activation segments, taken together with the other differences between CDK1–cyclin B and CDK2–cyclin A described above would be predicted to have significant consequences for the stability of the complexes that the two CDKs form with a cyclin subunit, their activation by phosphorylation and their ability to recognize a peptide substrate. These predictions are explored below. We hypothesize that the limited response of CDK1 to cyclin B binding is a consequence of CDK1 structural properties rather than the identity of the cyclin subunit: comparable studies of CDK2 show that it adopts an identical structure bound to either cyclin A^43^ or cyclin B^49^.

### Stability and activity of CDK1–cyclin B

**Figure 4:**
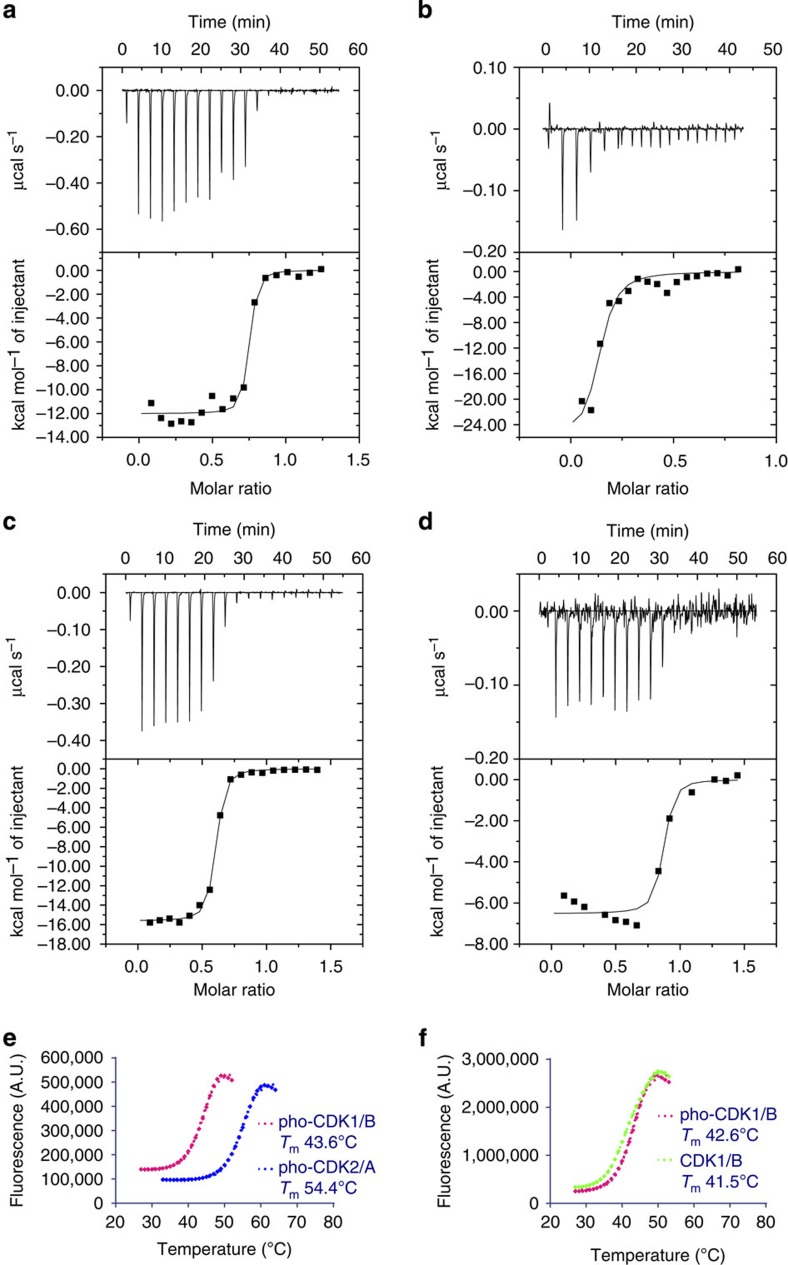
***In vitro*** **stability of the CDK1–cyclin B and CDK2–cyclin A complexes.** (**a**–**d**) Isothermal titration calorimetry thermograms to assess the formation of CDK2–cyclin A (**a**,**d**) and CDK1–cyclin B (**b**,**c**) complexes under conditions of lower salt, lower pH (40 mM HEPES, pH 7.4, 200 mM NaCl, 0.01% monothioglycerol) (**a**,**b**) and higher salt, higher pH (50 mM HEPES, pH 8.0, 500 mM NaCl, 0.01% monothioglycerol) (**c**,**d**). (**e**) Comparison of the thermal stability of the phosphorylated CDK1–cyclin B and CDK2–cyclin A complexes as assessed by differential scanning fluorimetry. (**f**) Comparison of the thermal stability of phosphorylated CDK1–cyclin B after either treatment with *λ*-phosphatase, or mock-treatment under identical conditions. (**e**,**f**) Representative melting curves are shown from two replicates.

The smaller interface between CDK1 and cyclin B would be expected to result in a decreased stability *in vitro*. To quantify the relative stability of unphosphorylated CDK1–cyclin B and unphosphorylated CDK2–cyclin A, we undertook isothermal titration calorimetry experiments. A direct comparison was confounded by the different preferred buffer conditions of the constituent proteins, whereas the binding of CDK2 to cyclin A could be evaluated in near physiological conditions (pH=7.4, 200 mM NaCl, T=30 °C), yielding a *K*_d_ value of 21 nM (Fig. 4a), cyclin B was prone to precipitation under these conditions, so that the thermogram accompanying its binding to CDK1 was hard to interpret quantitatively (Fig. 4b). Conversely, the binding of CDK1 to cyclin B could be analysed under higher salt, higher pH conditions (pH=8.0, 500 mM NaCl, *T*=30 °C), yielding a *K*_d_ value of 28 nM (Fig. 4c), but these conditions were not favourable for studying the binding of CDK2 to cyclin A (Fig. 4d). These experiments confirm that both unphosphorylated CDK2–cyclin A and unphosphorylated CDK1–cyclin B complexes can form with nanomolar affinity, but that this tight binding in the case of CDK1–cyclin B can only be observed under conditions of high salt.

To determine whether this difference in stability extends to the phosphorylated CDK1–cyclin B and CDK2–cyclin A complexes, we compared their stability at a consistent salt concentration (200 mM NaCl), using differential scanning fluorimetry (Fig. 4e). These experiments reveal that CDK2–cyclin A is substantially more thermally stable than CDK1–cyclin B (difference in melting temperature is *circa* 11 °C). This stability will depend on both the intrinsic thermal stability of the constituent proteins, and a contribution from the binding energy of the CDK for its cognate cyclin under the assay conditions.

To determine whether the thermal stability of CDK1–cyclin B complexes depends on the phosphorylation of the protein, we measured the melting temperature of phosphorylated CDK1–cyclin B after incubation with either *λ* phosphatase or buffer (Fig. 4f). A small (*circa* 1.0 °C) but reproducible difference in melting temperature was observed for the phosphatase-treated relative to the mock-treated sample, suggesting that phosphorylated CDK1–cyclin B is measurably more stable than its dephosphorylated form.

**Figure 5:**
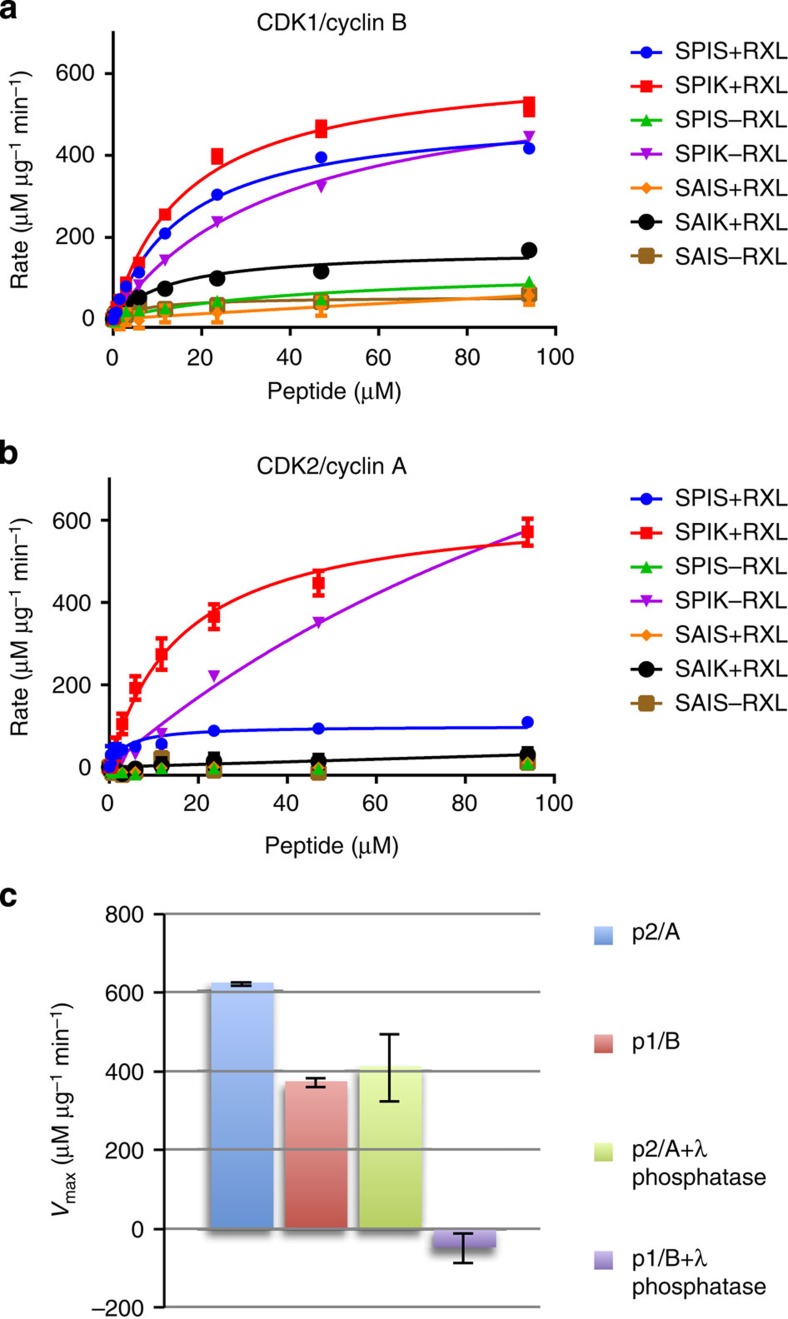
CDK1–cyclin B and CDK2–cyclin A activity. Seven peptide substrates derived from the sequence of p107 that systematically differ in the residues occupying the P+1 and P+3 sites (where P is the site of phospho-transfer) and whether the KRXL motif that binds to the cyclin recruitment site is present or absent was tested as substrates of CDK1–cyclin B (**a**) or CDK2–cyclin A (**b**). Two independent experiments were conducted with duplicate measurements for each point. Error bars on the observed data correspond to the range of observed values. The legend for each line indicates the local sequence around the phosphorylated serine (SPIK, SPIS, SAIS or SAIK), and whether an RXL motif was present (sequence KRRL, denoted ‘+RXL’) or absent (sequence AAAA, denoted ‘−RXL’). (**c,d**) Table of *k*_cat_/*K*_m_, _peptide_ values derived from Michaelis–Menton fit to the data shown in (**a**,**b**), respectively. (**e**) Activity towards the optimal peptide (SPIK+RXL) was re-determined following incubation of CDK1–cyclin B and CDK2–cyclin A with and without *λ*-phosphatase. Error bars indicate the s.d. of two independent measurements.

To explore to the extent to which the structural differences between CDK1–cyclin B and CDK2–cyclin A are reflected in their catalytic activity, both complexes were assayed against a panel of seven peptides (Fig. 5, Table 2, Supplementary Table 1). The peptides, derived from the sequence of the retinoblastoma-related protein p107 (ref. 51), systematically explore the requirements for a proline and a positively charged residue at the P+1 and P+3 positions, respectively (where P is the site of phospho-transfer), and for a KRRL sequence (the RXL motif) recognized by the cyclin recruitment site.

CDK2–cyclin A and CDK1–cyclin B share some aspects of substrate motif dependence: both are most active against substrates that have a canonical phosphorylation site and an intact RXL motif (peptide SPIK+RXL, Fig. 5a,b, Table 2); neither phosphorylate substrates that are highly compromised in the phosphorylation site (peptides SAIS-RXL and SAIS+RXL, Fig. 5a,b, Table 2); and, in the absence of an RXL motif, both phosphorylate peptides that have canonical phosphorylation sites (peptide SPIK-RXL, Fig. 5a,b, Table 2).

The two complexes do, however, display some differences in their phosphorylation of non-optimal substrates: where the RXL motif is retained, CDK2–cyclin A requires a proline at position P+1 (active against peptide SPIS+RXL), whereas CDK1–cyclin B shows activity against substrates that contain either proline at position P+1 (peptide SPIS+RXL) or lysine at position P+3 (peptide SAIK+RXL); in the absence of an RXL motif, CDK1 retains some activity against a substrate with an impaired phosphorylation site (peptide SPIS-RXL), whereas CDK2–cyclin A is essentially inactive against this substrate. Overall, therefore, CDK1 appears to be more accommodating of sub-optimal substrate sequences around the site of phosphotransfer.

Taken together the CDK1–cyclin B structure and the activity measurements suggest that the CDK1 activation segment is relatively flexible and we hypothesized that this flexibility might also be present in the T161-phosphorylated CDK1–cyclin B complex. To test this hypothesis we incubated phosphorylated CDK2–cyclin A and phosphorylated CDK1–cyclin B with *λ*-phosphatase. We have previously shown that susceptibility to dephosphorylation by *λ* phosphatase depends on details of the structure^44^: phosphorylated T160 of CDK2–cyclin A is buried in a cluster of positively charged residues and is therefore relatively refractory to dephosphorylation by *λ*-phosphatase, whereas phosphorylated T172 of CDK4 is within a flexible part of the activation segment and so can be readily dephosphorylated. As shown in Fig. 5c, there is a significant decrease in CDK1–cyclin B activity towards the peptide SPIK+RXL following incubation with *λ*-phosphatase under conditions where the CDK2–cyclin A activity is minimally affected. This result supports a model in which the activation segment of CDK1–cyclin B retains localized flexibility around T161 even when T161 is phosphorylated. We hypothesize that this local flexibility explains the relaxed substrate specificity of CDK1–cyclin B relative to CDK2–cyclin A described above.

A difference in CDK–cyclin complex stability has been previously observed in a study in which assembly of CDK2–cyclin A but not CDK1–cyclin B could be detected in cells in which CDK-activating kinase activity had been selectively inhibited^52^. CDK1 has been reported to also require activation segment phosphorylation to form a stable complex with cyclin A *in vitro*^53^ and *in vivo*^54^. From these observations it was proposed that stable association of cyclin B and CDK1 and activation segment phosphorylation are mutually dependent events. Our results support this model, since we find that the unphosphorylated CDK1–cyclin B complex requires supra-physiological salt concentrations for stability. We hypothesize that CDK2’s more extensive interaction with cyclin A and with cyclin B give it a competitive advantage over CDK1 for binding to either cyclin. We further hypothesize that CDK1 can redress this balance when the interaction can be re-enforced by phosphorylation of CDK1 on its activation segment^52^,^55^, a model that has been previously proposed from studies of CDK1 in crude cell extracts^55^.

### Structure of CDK1–cyclin B-Cks2-Compound 23

To evaluate the potential of CDK1 as a therapeutic target for cancer treatment, tool compounds that can selectively inhibit CDK1 are required. Distinguishing CDK1 and CDK2 by this means has proved to be difficult and CDK1 inhibitors described to date also show considerable activity against CDK2. For example, RO-3306, which is widely used as a selective CDK1 inhibitor (CDK1–cyclin B K_i_ 35 nM), is only 10-fold selective for CDK1–cyclin B over CDK2–cyclin E (K_i_ 340 nM)^37^. Structure-aided drug design has made a significant contribution to the development of selective and potent ATP-competitive protein kinase inhibitors, notably by revealing novel inhibitor binding modes as a result of kinase fold remodelling in response to inhibitor binding^56^,^57^.

**Figure 6:**
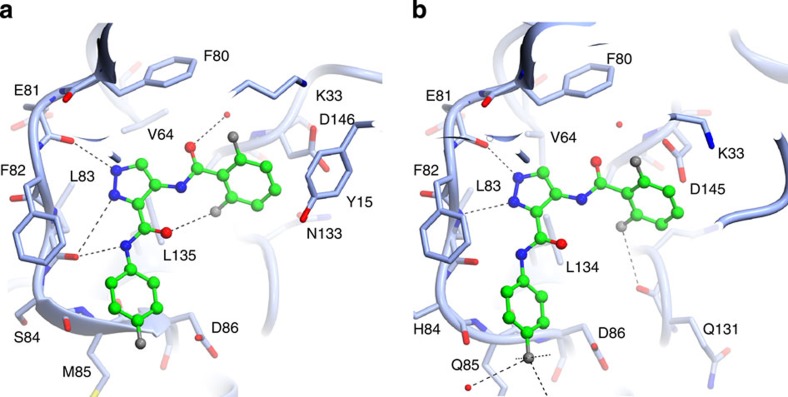
The structure of a CDK1–cyclin B-Cks2-ATP-competitive inhibitor complex. The binding modes of Compound 23 to CDK1 (**a**) and to CDK2 (PDB code 2VTP) (**b**) are compared. The structures of the two complexes are drawn in the same orientation and selected active site residues are included. The inhibitor is drawn in ball and stick mode with carbon atoms coloured green. Hydrogen bonds are rendered as dotted lines.

Therefore, we have also determined the structure of CDK1–cyclin B-Cks2 bound to Compound 23, a potent CDK1/CDK2 ATP-competitive inhibitor (IC_50_ CDK1, 10 nM; CDK2, 3 nM)^58^. This structure provides the first view of an ATP-competitive inhibitor bound within the CDK1 active site (Fig. 6). The presence of the inhibitor also improved the quality and resolution of the structure (Table 1). Overall a comparison of the apo- and inhibitor-bound CDK1–cyclin B-Cks2 complexes shows that inhibitor binding induces a slight rotation of the N-terminal β-sheet relative to the rest of the complex so that the glycine-rich loop folds down more effectively over the active site cleft (Fig. 6), and the anticipated salt-bridge between K33 and E51 is formed in the CDK1 active site. As expected, the pyrazole N2 of Compound 23 mimics the adenine N1 of ATP and accepts a hydrogen bond from the backbone amide of L83 while the pyrazole N1 and the amide nitrogen act as hydrogen bond donors to the carbonyl moieties of E81 and L83, respectively. The 4-fluorophenyl group is directed out of the active site cleft towards the surface of the C-terminal lobe so that the fluorine is pointing towards solvent and the side-chain of K89, and the aromatic ring makes a favourable π−π stacking interaction with the CDK1 backbone between M85 and D86. At the other end of the molecule, the 1,5 difluorophenyl ring forms an edge-face aromatic interaction with Y15 and occupies the active site behind the ribose binding site and overlapping with the α-phosphate-binding site. The residues that interact with Compound 23 are identical or highly conserved in the CDK1 and CDK2 sequences (Fig. 6) resulting in its almost equipotent activity towards the two CDKs.

Despite this degree of sequence identity, the observation that CDK1 remains relatively more flexible than CDK2 when cyclin-bound suggests possibilities to develop more potent CDK1-selective inhibitors. In particular, differences in the conformation of the activation segment impact recognition of ATP-competitive inhibitors and substitutions that exploit the local structural differences in this region might be anticipated to differentially affect binding to CDK1 and CDK2. Although challenging, the approach of selectively targeting kinases by exploiting their differing conformational plasticity is gaining acceptance^57^ and the availability of CDK1 and CDK2 structures provides a clinically relevant case to further test the hypothesis.

### CDK1–cyclin B and CDK2–cyclin A as CDK7 substrates

The CDK1 structures presented in this paper offer possible structural explanations for the observed differences in the activity of the CDK-activating kinase, CDK7–cyclin H towards monomeric CDK1 and CDK2, and CDK1 and CDK2–cyclin complexes^52^,^55^. CDK7–cyclin H has been reported to differentially recognize monomeric CDK1 and CDK2 and to phosphorylate monomeric CDK2 more efficiently than monomeric CDK1 (ref. 55). We observe that the CDK1 activation segment appears to be more flexible along its length than is the activation segment of CDK2, and that this flexibility renders residues near to the termini of the activation segment of CDK1 uninterpretable in our electron density maps, where equivalent residues of monomeric CDK2 adopt ordered conformations. We hypothesize that these residues, when suitably ordered, contribute to the recognition of CDK1 or CDK2 by CDK7–cyclin H. In support of this hypothesis, we note that cyclin binding both orders the terminal residues of the CDK1 activation segment and increases its susceptibility to phosphorylation by CDK7–cyclin H^52^,^59^. The increase in susceptibility to CDK7 that accompanies binding of CDK2 to cyclin A has been measured to be about 10-fold (*K*_m_ values for phosphorylation of CDK2 versus CDK2–cyclin A are *circa* 5.8 and 0.59 μΜ, respectively^55^).

Our analysis also suggests that, although the CDK1 and CDK2 activation segments are relatively well-ordered when bound to their cognate cyclins, they must retain some flexibility to access the CDK7 active site. A requirement for such flexibility is implicit in the non-identical positions of T160 (in CDK2–cyclin A) and T161 (in CDK1–cyclin B–Cks2): the Cα atoms of these residues are separated by 5 Å when the two structures are superimposed, so that one or both must move to engage productively with CDK7–cyclin H for phosphorylation.

Taken together, the data presented in this study are compatible with a model in which differences in stability and preferred conformation of the activation segments of different CDKs can be modulated by cyclin association. These effects could affect the extent of complex phosphorylation by changing the efficiency of the complex as a substrate of both activating kinases and inactivating phosphatases: the enhanced susceptibility of CDK1–cyclin B to dephosphorylation that we have demonstrated *in vitro* using *λ*-phosphatase may contribute to the relative dependence of CDK1–cyclin B and CDK2–cyclin A on CDK7–cyclin H. Differential dependence of CDK–cyclin complexes on levels of CDK7–cyclin H activity has been described elsewhere, based on cell-based studies^52^,^55^,^60^.

In summary, data presented in this paper reveal a number of important aspects of CDK1 structure and function. First they confirm the conserved nature of the inactive monomeric CDK fold and its ability to be remodelled. In the context of previously reported CDK–cyclin structures, the structure of CDK1–cyclin B increases the observed diversity with which the CDK responds to cyclin binding. Second, the CDK1–cyclin B structures reveal potential novel protein interaction sites that might regulate CDK1 activity. In addition to a conserved surface on the CDK1 C-terminal fold, there is a cleft between the two subunits not seen in the structure of CDK2–cyclin A but apparent in CDK9–cyclin T, where it is exploited by the human immunodeficiency virus 1 Tat^61^. Finally, we have shown that CDK1–cyclin B and CDK2–cyclin A, though closely related in sequence, can demonstrate differential dependence on sequence motifs in selecting their respective substrates. CDK1–cyclin B demonstrates a relatively relaxed specificity for residues adjacent to the site of phosphorylation, a phenomenon that correlates with this CDK–cyclin pair appearing to have a less rigidly constrained activation segment.

## Methods

### Protein expression and purification

*CDK, cyclin and Cks proteins*. Full-length human CDK1 was expressed in insect cells either untagged (from pVL1393) or as a 3C-protease cleavable GST fusion, which leaves a short cloning artefact (GPLGS) at the N terminus. To prepare the GST fusion, the CDK1 sequence was initially cloned into pGEX6P-1 as a Sma1-EcoR1 fragment. A cassette to express the fusion protein was extracted from recombinant pGEX6P-1 and cloned into the transfer vector pVL1393 and verified by DNA sequencing. Recombinant virus was generated using Sf9 cells co-transfected with pVL1393GST-CDK1 and FlashBacGold DNA (Oxford Expression Technologies) mixed with transfection reagent (GeneJuice, Novagen) and grown in SF900 II SFM media (LifeTechnologies). CDK1 expression was subsequently optimized by screening different virus stock:cell ratios. To purify GST-CDK1, cell pellets were resuspended in mTBS buffer (50 mM Tris-HCl, 150 mM NaCl, 1 mM dithiothreitol (DTT), pH7.5) supplemented with 200 μM RNAase A, 200 μM DNAaseI and 200 μM MgCl_2_, lysed by sonication then clarified by centrifugation (60,000 × *g*, 1 h). GST-CDK1 was subsequently purified by sequential affinity chromatography (glutathione-sepharose 4B, GE Healthcare), GST-3C cleavage (1:50 w/w at +4 °C overnight) and a final size-exclusion chromatography (SEC) step (Superdex 75 26/60 column, GEHealthcare equilibrated in mTBS). Human cyclin B1, residues 165–433 carrying the C167S/C238S/C350S mutations, was expressed in recombinant *E. coli* cells and purified as described exploiting the thrombin-cleavable hexa-histidine tag encoded by the pET28-(a+) vector^45^. This strategy leaves a GSHM artefact N-terminal to N165. Human Cks1 and Cks2 were expressed as His_6_-or GST-tagged proteins, respectively, and purified by sequential affinity (Ni-NTA or glutathione-sepharose 4B), 3C cleavage (in the case of the GST fusion) and SEC steps. To prepare the CDK1–Cks1 complex, purified His-tagged Cks1 was immobilized on Ni-NTA and used to capture untagged CDK1 from insect cell lysate. The complex was eluted with imidazole and further purified by SEC on a HR26/60 SD75 column (GE Healthcare) equilibrated in HBS (10 mM HEPES pH 7.5/150 mM NaCl/0.01% monothioglycerol (MTG)/0.01% sodium azide). To prepare the ternary CDK1–cyclin B-Cks2 complex, components were individually purified and then mixed in a molar ratio of 1:1.5:2, CDK1:cyclin B:Cks2. The interaction between CDK1 and cyclin B has been reported to be stable *in vitro*^53^, but we observed that it is sensitive to buffer conditions. Following optimization of the salt concentration, the final step to assemble the complex was carried out on a Superdex 75 HR26/60 size-exclusion column equilibrated in a modified Tris-buffered saline containing 1.0 M NaCl, 50 mM Tris-HCl, pH 8.0, 1 mM DTT. Fractions containing the desired complexes were pooled, and both were concentrated to circa 10–12 mg ml^−1^ by ultrafiltration. Both the CDK1–Cks1 and CDK1–cyclin B–Cks2 complexes can be successfully crystallized from frozen samples. Proteins were concentrated to circa 10–12 mg ml^−1^ and then fast frozen in aliquots in liquid nitrogen before storage at −80 °C.

To prepare fully phosphorylated CDK1–cyclin B for kinase assays, monomeric CDK1 was first phosphorylated by *S. cerevisiae* CAK1 *in vitro*. GST-CAK1 was expressed and purified from recombinant *E. coli* cells as described^62^. Purified CDK1 and GST-CAK1 were mixed in TBS buffer (50 mM Tris-Cl, 200 mM NaCl, 0.01% MTG, pH7.5) supplemented with 1 mM ATP and 10 mM MgCl_2_ and incubated overnight at 4 °C. The extent of CDK1 phosphorylation was determined by an HPLC assay (described below and Supplementary Fig. 5) by which criteria the CDK1 was judged to be circa 100% phosphorylated. The GST-CAK1 and CDK1 were separated by SEC using a Superdex 200 16/60 column, and then the CDK1–cyclin B complex was prepared by mixing the CDK1 with excess cyclin B and subsequent purification by SEC as described above for preparation of the CDK1–cyclin B–Cks2 complex.

To prepare unphosphorylated CDK1 for isothermal titration calorimetry experiments, purified monomeric CDK1 (1 mg) was incubated with *λ*-phosphatase (400 units) in a total volume of 1 ml in TBS buffer with the supplements for the *λ*-phosphatase reaction (1 × NEB PMP Supplement, 1 mM MnCl_2_, 50 mM Tris-Cl, 200 mM NaCl, 0.01% MTG, pH7.5). The reaction was carried out at 30 °C for 30 min and CDK1 was subsequently purified by SEC using a Superdex 75 16/60 column. The extent of CDK1 dephosphorylation was determined by an HPLC assay (described below and Supplementary Fig. 5) and judged to be circa 100% complete.

To prepare unphosphorylated CDK1–cyclin B for differential scanning fluorimetry experiments, purified phosphorylated CDK1–cyclin B (69 μg) was incubated, half with *λ*-phosphatase (64 units, phosphatase-treated sample) and half with buffer (mock-treated sample), each in a total volume of 34 μl in TBS buffer with the supplements for the *λ*-phosphatase reaction (1 × NEB PMP Supplement, 1 mM MnCl_2_, 50 mM Tris-Cl, 200 mM NaCl, 0.01% MTG, pH7.5). The reaction was carried out at 30 °C for 30 min.

Bovine cyclin A^63^, human CDK2^64^ and T160-phosphorylated CDK2–cyclin A^50^ were prepared as described. In brief cyclin A and CDK2 were purified by sequential affinity and SEC steps exploiting, respectively, His_6_ and GST tags. CDK2–cyclin A was assembled by first purifying GST-CDK2 on a glutathione affinity column and then using the modified column to affinity purify untagged cyclin A. The glutathione eluate was then incubated with 3C protease to release the CDK2–cyclin A from GST, which was subsequently further purified by SEC and glutathione affinity chromatography to remove any co-eluting GST.

### Substrate peptides

DNA sequences were gene-synthesized (GenScript) to express a set of eight CDK substrate peptides that differ in sequence at the P+1 and P+3 residues (where P is the site of phospho-transfer) and at the RXL sequence that binds to the cyclin recruitment site. The peptides are based on a previously described set derived from the sequence of p107 (ref. 51). However, they differ in that they include peptides in which the P+1 proline is substituted by an alanine and Y648 and I674 are each substituted by a tryptophan to permit accurate determination of the peptide concentration by measuring the optical density at 280 nm. In addition, so that there is only one SP motif in the sequence, P651 was mutated to alanine. The sequences were subsequently ligated into pGEX6P-1 cut with *Bam*H1 and *Eco*R1 by ligation-independent cloning and verified by sequencing. The peptides were expressed in recombinant *E. coli* Rosetta(DE3)/pLysS cells at 20 °C overnight subsequent to induction with 0.1 mM IPTG and then purified by affinity chromatography and a second ion-exchange chromatography step using either HiTrap Q FF 5 ml or HiTrap SP FF 5 ml columns (GE Healthcare) equilibrated into 25 mM MES pH 6.5. Peptides were loaded onto the appropriate column (dependent on their pI) and then stepped into 25 mM MES, 0.5M NaCl, pH6.5 for peptide rapid release. Fractions containing the peptides were confirmed by sodium dodecyl sulfate–PAGE (SDS–PAGE), pooled, concentrated to a final peptide concentration between 1 and 2 mg ml^−1^ and then flash frozen and stored at −80 °C in 100 μl aliquots. Peptide concentrations were determined from calculated extinction coefficients (http://web.expasy.org/protparam/) using a Nanodrop2000 (Thermoscientific).

### HPLC analysis of CDK1

The extent of CDK1 phosphorylation (either as purified from recombinant insect cells or subsequent to phosphorylation *in vitro* by GST-CAK1) was monitored by HPLC. CDK1 (0.1 ml) was injected onto a Jupiter 5 u C4 300A column (Phenomenex) equilibrated in 0.01% TFA in H_2_O (Buffer A) and then a 0.01% TFA/acetonitrile (buffer B) gradient was developed from 50 to 60% buffer B over 20 column volumes. Peaks of phosphorylated and unphosphorylated CDK1 are well resolved (Supplementary Fig. 5). The identity of the CDK1 species in each peak was confirmed by mass spectrometry.

### CDK kinase assays

Phosphorylated CDK2–cyclin A and CDK1–cyclin B were assayed against a panel of seven peptides using the ADP-Glo assay (Promega) essentially as described by the manufacturers. In brief, in a final volume of 5 μl, reactions were carried out at room temperature in 40 mM Tris-HCl pH 7.5, containing 20 mM MgCl_2_, 0.1 mg ml^−1^ BSA, using 4 nM CDK1-cyclinB or 1.5 nM CDK2–cyclin A. Peptide dilutions (94 to 0 μM) were added to the CDK–cyclin solution and the reactions were initiated by the addition of 75 μM ATP. All activity assays were performed in duplicate in white low volume 384-well plates using a PheraStar plate reader (BMG). *K*_m_ and *V*_max_ values were obtained by fitting the data to the equation *v*=*V*_max_/(1+(*K*_m_/[*S*])) using PRISM (GraphPad).

To characterize their susceptibility to dephosphorylation by *λ*-phosphatase, phosphorylated CDK2–cyclin A (2.5 nM) and phosphorylated CDK1–cyclin B (4 nM) were incubated with *λ*-phosphatase (32 units), supplemented with 400 μM MnCl_2_ for 30 min at 30 °C in a total volume of 60 μl. As the positive controls, CDK1–cyclin B and CDK2–cyclin A were mock incubated with buffer only. All four samples were then assayed using the SPIK+RXL peptide in the ADP-Glo assay format as described above.

### Isothermal titration calorimetry

Protein samples were exchanged into matching buffers using a HiTrap desalting column (GE Healthcare Life Sciences). Two buffer conditions were used, 50 mM HEPES pH 8.0, 500 mM NaCl, 0.01% MTG and 40 mM HEPES, 200 mM NaCl, 0.01% MTG, pH7.4. Measurements were conducted on a MicroCal ITC 200 (Malvern Instruments). Human cyclin B and bovine cyclin A2 were used as samples in the cell and unphosphorylated CDK1 and CDK2 were used as ligands. Because human cyclin A2 is very susceptible to aggregation, bovine cyclin A2 is routinely used as a surrogate for studies requiring monomeric cyclin A. The bovine cyclin A2 construct (residues 169–430)^63^ corresponds to residues 171–432 in the human cyclin A2 sequence. Protein concentrations were measured by *A*_280 nm_ on a Nanodrop2000 (Thermoscientific) using calculated extinction coefficients (http://web.expasy.org/protparam/). Titrations were carried out at 30 °C. The stability of the individual proteins confounded a number of experiments and considerable optimization was required. The presented thermograms were collected under the following conditions: In lower salt and lower pH buffer (40 mM HEPES, 200 mM NaCl, 0.01% MTG, pH7.4) preferred by CDK2–cyclin A: cyclin B and cyclin A were in the cell at 60 and 200 μM, and CDK1 and CDK2 were added from the syringe at 18 and 30 μM, respectively. In the higher salt, higher pH buffer conditions (50 mM HEPES pH 8.0, 500 mM NaCl, 0.01% MTG) preferred by CDK1–cyclin B: cyclin B and cyclin A were both in the cell at 140 μM, and CDK1 and CDK2 were added at 16 and 18 μM, respectively. Origin 7.0 (OriginLab Corp.) was used for all data analysis, including ligand dilution subtractions and data fitting to the one-set-of-sites model.

### Differential scanning fluorimetry

Thermal melting experiments were carried out using a ViiA7 Real-Time PCR system (Applied Biosystems). Purified phosphorylated CDK1–cyclin B (4 μM, in 50 mM Tris-HCl, pH 8.0, 200 mM NaCl, 0.01% MTG) and phosphorylated CDK2–cyclin A (6 μM, in 40 mM HEPES, pH 7.0, 200 mM NaCl) were assayed, in triplicate, in a 384-well plate. For experiments to assess the effect of phosphatase treatment on complex stability, samples were prepared as described above. SyPRO Orange (× 10) (Invitrogen) was used as the fluorescent probe, and fluorescence was measured using the ROX reporter channel (excitation *λ*=470 nm, emission *λ*=586 nm). Complex stability was assessed by increasing the temperature from 25 to 95 °C through 1 °C increments. The resulting data were plotted and the inflection point (*T*_m_) calculated using the Boltzmann equation in PRISM (GraphPad).

### Crystallization, data collection and structure determination

Initial crystallization conditions for both complexes were identified following screening against a wide range of commercially available screens. Typically drops at a 1:1 or 2:1 protein:well solution ratios (200 or 300 nl total volume, respectively, protein solution concentration 10–12 mg ml^−1^) were set up using a Mosquito robot (TTP Labtech) and incubated at 4 °C. Single crystals of CDK1–Cks1 were identified in 16–18% PEG10K, 0.1 M imidazole pH 8.2–8.4. CDK1–cyclin B–Cks2 crystallization conditions were optimized from a Morpheus screen condition B4 hit (Molecular Dimensions) to 0.1 M MES/imidazole buffer (pH 6.7), 6.5% MPD, 5% PEG4K, 10% PEG1K. The CDK1–Cks1 crystals were cryo-protected in the crystallization solution supplemented with ethylene glycol, whereas the CDK1–cyclin B-Cks2 crystals grow from a cryo-protecting solution. Crystals of the CDK1–cyclin B-Cks2-Compound 23 complex were grown by adding Compound 23 in twofold molar excess (which was also a >10-fold concentration than the inhibitor IC_50_) to a low concentration solution of CDK1–cyclin B-Cks2 and then concentrating the sample to 10–12 mg ml^−1^ for crystallization. Crystals were flash cooled in liquid nitrogen before data collection. Data processing was carried out using XDS, MOSFLM, POINTLESS/AIMLESS^65^ and other programs of the CCP4 suite^66^, run through the CCP4i2 GUI. The structures of the different complexes were solved by molecular replacement using Phaser^67^, and search models drawn from PDB entries 1BUH (Cks2), 1HCK (monomeric CDK2), 2B9R (human cyclin B) and 1QMZ (cyclin-bound CDK2). Structures were refined using REFMAC^68^, interspersed with manual rebuilding in Coot^69^, including TLS refinement. The statistics for the data sets and for the crystallographic refinement are presented in Table 1. Representative examples of the refined 2F_o-_F_c_ electron density maps associated with each structure are shown in Supplementary Fig. 6.

## Additional information

**Accession codes:** The structures of CDK1–Cks1, CDK1–cyclin B–Cks2 and CDK1–cyclin B–Cks2-Compound 23 and their associated structure factors have been deposited in the PDB with accession codes 4YC6, 4YC3 and 4Y72, respectively.

**How to cite this article:** Brown, N. R. *et al*. CDK1 structures reveal conserved and unique features of the essential cell cycle CDK. *Nat. Commun.* 6:6769 doi: 10.1038/ncomms7769 (2015).

## Supplementary Material

Supplementary Information Supplementary Figures 1-6, Supplementary Table 1 and Supplementary References

## Figures and Tables

**Table 1:** X-ray data collection and refinement statistics.

	**CDK1–Cks1**	**CDK1–cyclin B-Cks2**	**CDK1–cyclin B-Cks2-Compound 23**
Data collection			
Space group	P2_1_	P2_1_2_1_2_1_	P2_1_2_1_2_1_
Cell dimensions			
*a*, *b*, *c* (Å)	66.8, 147.5, 87.3	69.2, 70.2, 156.2	65.2, 68.7, 166.8
*α*, *β*, *γ* (°)	90.0, 92.1, 90.0	90.0, 90.0, 90.0	90.0, 90.0, 90.0
Resolution(Å)	75.10-2.80 (2.91-2.80)	52.19-2.80 (2.95-2.80)	53.00-2.30 (2.38-2.30)
Rmerge	0.08(0.89)	0.15 (0.57)	0.09 (0.90)
I/σ(I)	15.3(1.8)	7.2 (2.0)	13.7 (2.4)
Completeness (%)	99.7 (98.8)	99.6 (97.9)	99.9 (99.9)
Redundancy	5.9 (5.2)	3.6 (3.2)	6.7 (6.4)
			
Refinement			
Resolution(Å)	75.10-2.80	78.10-2.80	53.00-2.30
No. reflections	41,491 (1,787)	19,278 (1,014)	34,010 (1,697)
R_work_/R_free_	0.234 (0.269)	0.209 (0.282)	0.200 (0.254)
No. atoms			
Protein	23,200	10,377	10,291
Ligand/ion	0	0	37
Water	0	33	101
B-factors			
Protein	36.2	39.2	24.2
Ligand/ion	NA	NA	38.0
Water	NA	14.8	38.7
R.m.s.d.			
Bond lengths (Å)	0.012	0.013	0.013
Bond angles (°)	1.679	1.696	1.687

NA, not applicable; r.m.s.d., root mean-squared deviations.

**Table 2:** *k*_cat_/*K*_m, peptide_ (s^−1^) for CDK–cyclin complexes on model substrates.

**Phosphorylated CDK1/cyclin B**					**Phosphorylated CDK2/cyclin A**				
	**SPIK**	**SAIK**	**SPIS**	**SAIS**		**SPIK**	**SAIK**	**SPIS**	**SAIS**
+RXL	0.52	0.17	0.42	0.06	+RXL	0.57	0.03	0.11	0.01
−RXL	0.45	ND*	0.09	0.06	−RXL	0.57	ND	0.01	0.01

^*^ND, not determined.

## References

[b1] Lohka M. J., Hayes M. K. & Maller J. L. Purification of maturation-promoting factor, an intracellular regulator of early mitotic events. Proc. Natl Acad. Sci. USA 85, 3009–3013 (1988) . 3283736 10.1073/pnas.85.9.3009 PMC280132

[b2] Draetta G., Brizuela L., Potashkin J. & Beach D. Identification of p34 and p13, human homologs of the cell cycle regulators of fission yeast encoded by cdc2+ and suc1+. Cell 50, 319–325 (1987) . 3297353 10.1016/0092-8674(87)90227-3

[b3] Lee M. G. & Nurse P. Complementation used to clone a human homologue of the fission yeast cell cycle control gene cdc2. Nature 327, 31–35 (1987) . 3553962 10.1038/327031a0

[b4] Morgan D. O. The cell cycle: principles of control Oxford University Press (2007) .

[b5] Pines J. & Hunter T. Human cyclin A is adenovirus E1A-associated protein p60 and behaves differently from cyclin B. Nature 346, 760–763 (1990) . 2143810 10.1038/346760a0

[b6] Giordano A. . A 60 kd cdc2-associated polypeptide complexes with the E1A proteins in adenovirus-infected cells. Cell 58, 981–990 (1989) . 2570639 10.1016/0092-8674(89)90949-5

[b7] Labib K. How do Cdc7 and cyclin-dependent kinases trigger the initiation of chromosome replication in eukaryotic cells? Genes Dev. 24, 1208–1219 (2010) . 20551170 10.1101/gad.1933010 PMC2885657

[b8] den Elzen N. & Pines J. Cyclin A is destroyed in prometaphase and can delay chromosome alignment and anaphase. J. Cell Biol. 153, 121–136 (2001) . 11285279 10.1083/jcb.153.1.121 PMC2185531

[b9] Geley S. . Anaphase-promoting complex/cyclosome-dependent proteolysis of human cyclin A starts at the beginning of mitosis and is not subject to the spindle assembly checkpoint. J. Cell Biol. 153, 137–148 (2001) . 11285280 10.1083/jcb.153.1.137 PMC2185534

[b10] Pines J. & Hunter T. Isolation of a human cyclin cDNA: evidence for cyclin mRNA and protein regulation in the cell cycle and for interaction with p34cdc2. Cell 58, 833–846 (1989) . 2570636 10.1016/0092-8674(89)90936-7

[b11] Deibler R. W. & Kirschner M. W. Quantitative reconstitution of mitotic CDK1 activation in somatic cell extracts. Mol. Cell 37, 753–767 (2010) . 20347419 10.1016/j.molcel.2010.02.023 PMC2882237

[b12] Pines J. & Hunter T. Human cyclins A and B1 are differentially located in the cell and undergo cell cycle-dependent nuclear transport. J. Cell Biol. 115, 1–17 (1991) . 1717476 10.1083/jcb.115.1.1 PMC2289910

[b13] Gavet O. & Pines J. Progressive activation of CyclinB1-Cdk1 coordinates entry to mitosis. Dev. Cell 18, 533–543 (2010) . 20412769 10.1016/j.devcel.2010.02.013 PMC3325599

[b14] Fung T. K. & Poon R. Y. A roller coaster ride with the mitotic cyclins. Semin. Cell Dev. Biol. 16, 335–342 (2005) . 15840442 10.1016/j.semcdb.2005.02.014

[b15] Lindqvist A., Rodriguez-Bravo V. & Medema R. H. The decision to enter mitosis: feedback and redundancy in the mitotic entry network. J. Cell Biol. 185, 193–202 (2009) . 19364923 10.1083/jcb.200812045 PMC2700378

[b16] Alvarez-Fernandez M. & Malumbres M. Preparing a cell for nuclear envelope breakdown: Spatio-temporal control of phosphorylation during mitotic entry. Bioessays 36, 757–765 (2014) . 24889070 10.1002/bies.201400040

[b17] Holt L. J. . Global analysis of Cdk1 substrate phosphorylation sites provides insights into evolution. Science 325, 1682–1686 (2009) . 19779198 10.1126/science.1172867 PMC2813701

[b18] Mochida S. & Hunt T. Protein phosphatases and their regulation in the control of mitosis. EMBO Rep. 13, 197–203 (2012) . 22482124 10.1038/embor.2011.263 PMC3323141

[b19] Clute P. & Pines J. Temporal and spatial control of cyclin B1 destruction in metaphase. Nat. Cell Biol. 1, 82–87 (1999) . 10559878 10.1038/10049

[b20] Peters J. M. The anaphase promoting complex/cyclosome: a machine designed to destroy. Nat. Rev. Mol. Cell Biol. 7, 644–656 (2006) . 16896351 10.1038/nrm1988

[b21] Barford D. Structural insights into anaphase-promoting complex function and mechanism. Philos. Trans. R. Soc. Lond. B. Biol. Sci. 366, 3605–3624 (2011) . 22084387 10.1098/rstb.2011.0069 PMC3203452

[b22] Malumbres M. & Barbacid M. Cell cycle, CDKs and cancer: a changing paradigm. Nat. Rev. Cancer 9, 153–166 (2009) . 19238148 10.1038/nrc2602

[b23] Satyanarayana A. . Genetic substitution of Cdk1 by Cdk2 leads to embryonic lethality and loss of meiotic function of Cdk2. Development 135, 3389–3400 (2008) . 18787066 10.1242/dev.024919 PMC2668819

[b24] Santamaria D. . Cdk1 is sufficient to drive the mammalian cell cycle. Nature 448, 811–815 (2007) . 17700700 10.1038/nature06046

[b25] Diril M. K. . Cyclin-dependent kinase 1 (Cdk1) is essential for cell division and suppression of DNA re-replication but not for liver regeneration. Proc. Natl Acad. Sci. USA 109, 3826–3831 (2012) . 22355113 10.1073/pnas.1115201109 PMC3309725

[b26] Adhikari D. . Cdk1, but not Cdk2, is the sole Cdk that is essential and sufficient to drive resumption of meiosis in mouse oocytes. Hum. Mol. Genet. 21, 2476–2484 (2012) . 22367880 10.1093/hmg/dds061

[b27] Hayles J., Beach D., Durkacz B. & Nurse P. The fission yeast cell cycle control gene cdc2: isolation of a sequence suc1 that suppresses cdc2 mutant function. Mol. Gen. Genet. 202, 291–293 (1986) . 3010051 10.1007/BF00331653

[b28] Patra D., Wang S. X., Kumagai A. & Dunphy W. G. The xenopus Suc1/Cks protein promotes the phosphorylation of G(2)/M regulators. J. Biol. Chem. 274, 36839–36842 (1999) . 10601234 10.1074/jbc.274.52.36839

[b29] Koivomagi M. . Multisite phosphorylation networks as signal processors for Cdk1. Nat. Struct. Mol. Biol. 20, 1415–1424 (2013) . 24186061 10.1038/nsmb.2706 PMC3855452

[b30] Koivomagi M. . Cascades of multisite phosphorylation control Sic1 destruction at the onset of S phase. Nature 480, 128–131 (2011) . 21993622 10.1038/nature10560 PMC3228899

[b31] Bourne Y. . Crystal structure and mutational analysis of the human CDK2 kinase complex with cell cycle-regulatory protein CksHs1. Cell 84, 863–874 (1996) . 8601310 10.1016/s0092-8674(00)81065-x

[b32] McGrath D. A. . Cks confers specificity to phosphorylation-dependent CDK signaling pathways. Nat. Struct. Mol. Biol. 20, 1407–1414 (2013) . 24186063 10.1038/nsmb.2707 PMC4242096

[b33] Cheng C. K. . Dual blockade of lipid and cyclin-dependent kinases induces synthetic lethality in malignant glioma. Proc. Natl Acad. Sci. USA 109, 12722–12727 (2012) . 22802621 10.1073/pnas.1202492109 PMC3411950

[b34] Johnson N. & Shapiro G. I. Cyclin-dependent kinases (cdks) and the DNA damage response: rationale for cdk inhibitor-chemotherapy combinations as an anticancer strategy for solid tumors. Expert Opin. Ther. Targets 14, 1199–1212 (2010) . 20932174 10.1517/14728222.2010.525221 PMC3957489

[b35] Johnson N. . Compromised CDK1 activity sensitizes BRCA-proficient cancers to PARP inhibition. Nat. Med. 17, 875–882 (2011) . 21706030 10.1038/nm.2377 PMC3272302

[b36] Cepeda D. . CDK-mediated activation of the SCF(FBXO) (28) ubiquitin ligase promotes MYC-driven transcription and tumourigenesis and predicts poor survival in breast cancer. EMBO Mol. Med. 5, 999–1018 (2013) . 10.1002/emmm.201202341 PMC3721474 23776131

[b37] Vassilev L. T. . Selective small-molecule inhibitor reveals critical mitotic functions of human CDK1. Proc. Natl Acad. Sci. USA 103, 10660–10665 (2006) . 16818887 10.1073/pnas.0600447103 PMC1502288

[b38] Bruyere C. & Meijer L. Targeting cyclin-dependent kinases in anti-neoplastic therapy. Curr. Opin. Cell Biol. 25, 772–779 (2013) . 24011867 10.1016/j.ceb.2013.08.004

[b39] Malumbres M., Pevarello P., Barbacid M. & Bischoff J. R. CDK inhibitors in cancer therapy: what is next? Trends Pharmacol. Sci. 29, 16–21 (2008) . 18054800 10.1016/j.tips.2007.10.012

[b40] Hole A. J. . Comparative structural and functional studies of 4-(thiazol-5-yl)-2-(phenylamino)pyrimidine-5-carbonitrile CDK9 inhibitors suggest the basis for isotype selectivity. J. Med. Chem. 56, 660–670 (2013) . 23252711 10.1021/jm301495v PMC3579457

[b41] Cho Y. S. . Fragment-based discovery of 7-azabenzimidazoles as potent, highly selective, and orally active CDK4/6 inhibitors. ACS Med. Chem. Lett. 3, 445–449 (2012) . 24900493 10.1021/ml200241a PMC4025827

[b42] De Bondt H. L. . Crystal structure of cyclin-dependent kinase 2. Nature 363, 595–602 (1993) . 8510751 10.1038/363595a0

[b43] Jeffrey P. D. . Mechanism of CDK activation revealed by the structure of a cyclinA-CDK2 complex. Nature 376, 313–320 (1995) . 7630397 10.1038/376313a0

[b44] Takaki T. . The structure of CDK4/cyclin D3 has implications for models of CDK activation. Proc. Natl Acad. Sci. USA 106, 4171–4176 (2009) . 19237555 10.1073/pnas.0809674106 PMC2657433

[b45] Petri E. T., Errico A., Escobedo L., Hunt T. & Basavappa R. The crystal structure of human cyclin B. Cell Cycle 6, 1342–1349 (2007) . 17495533 10.4161/cc.6.11.4297

[b46] Russo A. A., Jeffrey P. D., Patten A. K., Massague J. & Pavletich N. P. Crystal structure of the p27Kip1 cyclin-dependent-kinase inhibitor bound to the cyclin A-Cdk2 complex. Nature 382, 325–331 (1996) . 8684460 10.1038/382325a0

[b47] Day P. J. . Crystal structure of human CDK4 in complex with a D-type cyclin. Proc. Natl Acad. Sci. USA 106, 4166–4170 (2009) . 19237565 10.1073/pnas.0809645106 PMC2657441

[b48] Baumli S. . The structure of P-TEFb (CDK9/cyclin T1), its complex with flavopiridol and regulation by phosphorylation. EMBO J. 27, 1907–1918 (2008) . 18566585 10.1038/emboj.2008.121 PMC2486423

[b49] Brown N. R. . Cyclin B and cyclin A confer different substrate recognition properties on CDK2. Cell Cycle 6, 1350–1359 (2007) . 17495531 10.4161/cc.6.11.4278

[b50] Brown N. R., Noble M. E., Endicott J. A. & Johnson L. N. The structural basis for specificity of substrate and recruitment peptides for cyclin-dependent kinases. Nat. Cell Biol. 1, 438–443 (1999) . 10559988 10.1038/15674

[b51] Leng X., Noble M., Adams P. D., Qin J. & Harper J. W. Reversal of growth suppression by p107 via direct phosphorylation by cyclin D1/cyclin-dependent kinase 4. Mol. Cell Biol. 22, 2242–2254 (2002) . 11884610 10.1128/MCB.22.7.2242-2254.2002 PMC133692

[b52] Larochelle S. . Requirements for Cdk7 in the assembly of Cdk1/cyclin B and activation of Cdk2 revealed by chemical genetics in human cells. Mol. Cell 25, 839–850 (2007) . 17386261 10.1016/j.molcel.2007.02.003 PMC1858677

[b53] Desai D., Wessling H. C., Fisher R. P. & Morgan D. O. Effects of phosphorylation by CAK on cyclin binding by CDC2 and CDK2. Mol. Cell Biol. 15, 345–350 (1995) . 7799941 10.1128/mcb.15.1.345 PMC231966

[b54] Larochelle S., Pandur J., Fisher R. P., Salz H. K. & Suter B. Cdk7 is essential for mitosis and for *in vivo* Cdk-activating kinase activity. Genes Dev. 12, 370–381 (1998) . 9450931 10.1101/gad.12.3.370 PMC316490

[b55] Merrick K. A. . Distinct activation pathways confer cyclin-binding specificity on Cdk1 and Cdk2 in human cells. Mol. Cell 32, 662–672 (2008) . 19061641 10.1016/j.molcel.2008.10.022 PMC2643088

[b56] Noble M. E., Endicott J. A. & Johnson L. N. Protein kinase inhibitors: insights into drug design from structure. Science 303, 1800–1805 (2004) . 15031492 10.1126/science.1095920

[b57] Zhao Z. . Exploration of type II binding mode: a privileged approach for kinase inhibitor focused drug discovery? ACS Chem. Biol. 9, 1230–1241 (2014) . 24730530 10.1021/cb500129t PMC4068218

[b58] Wyatt P. G. . Identification of N-(4-piperidinyl)-4-(2,6-dichlorobenzoylamino)-1H-pyrazole-3-carboxamide (AT7519), a novel cyclin dependent kinase inhibitor using fragment-based X-ray crystallography and structure based drug design. J. Med. Chem. 51, 4986–4999 (2008) . 18656911 10.1021/jm800382h

[b59] Fisher R. P. & Morgan D. O. A novel cyclin associates with MO15/CDK7 to form the CDK-activating kinase. Cell 78, 713–724 (1994) . 8069918 10.1016/0092-8674(94)90535-5

[b60] Schachter M. M. . A Cdk7-Cdk4 T-loop phosphorylation cascade promotes G1 progression. Mol. Cell 50, 250–260 (2013) . 23622515 10.1016/j.molcel.2013.04.003 PMC3677717

[b61] Tahirov T. H. . Crystal structure of HIV-1 Tat complexed with human P-TEFb. Nature 465, 747–751 (2010) . 20535204 10.1038/nature09131 PMC2885016

[b62] Brown N. R. . Effects of phosphorylation of threonine 160 on cyclin-dependent kinase 2 structure and activity. J. Biol. Chem. 274, 8746–8756 (1999) . 10085115 10.1074/jbc.274.13.8746

[b63] Brown N. R. . The crystal structure of cyclin A. Structure 3, 1235–1247 (1995) . 8591034 10.1016/s0969-2126(01)00259-3

[b64] Welburn J. & Endicott J. Methods for preparation of proteins and protein complexes that regulate the eukaryotic cell cycle for structural studies. Methods Mol. Biol. 296, 219–235 (2005) . 15576935 10.1385/1-59259-857-9:219

[b65] Evans P. An introduction to data reduction: space-group determination, scaling and intensity statistics. Acta Crystallogr. Sect. D. D67, 282–292 (2011) . 10.1107/S090744491003982X PMC3069743 21460446

[b66] CCP4. The CCP4 suite: programs for protein crystallography. Acta Crystallogr. D. Biol. Crystallogr. 50, 760–763 (1994) . 15299374 10.1107/S0907444994003112

[b67] McCoy A. J. . Phaser crystallographic software. J. Appl. Crystallogr. 40, 658–674 (2007) . 19461840 10.1107/S0021889807021206 PMC2483472

[b68] Murshudov G. N., Vagin A. A. & Dodson E. J. Refinement of macromolecular structures by the maximum-likelihood method. Acta Crystallogr. D. Biol. Crystallogr. 53, 240–255 (1997) . 15299926 10.1107/S0907444996012255

[b69] Emsley P., Lohkamp B., Scott W. G. & Cowtan K. Features and development of Coot. Acta Crystallogr. D. Biol. Crystallogr. 66, 486–501 (2010) . 20383002 10.1107/S0907444910007493 PMC2852313

[b70] McNicholas S., Potterton E., Wilson K. S. & Noble M. E. Presenting your structures: the CCP4mg molecular-graphics software. Acta Crystallogr. D. Biol. Crystallogr. 67, 386–394 (2011) . 21460457 10.1107/S0907444911007281 PMC3069754

