# Supplementary material for: CDK1 structures reveal conserved and unique features of the essential cell cycle CDK
Source: Nat Commun. 2015 Apr 13;6:6769. doi: 10.1038/ncomms7769 (PMC4413027; doi:10.1038/ncomms7769)
Supplement: Supplementary Information — Supplementary Figures 1-6, Supplementary Table 1 and Supplementary References [file ncomms7769-s1.pdf]

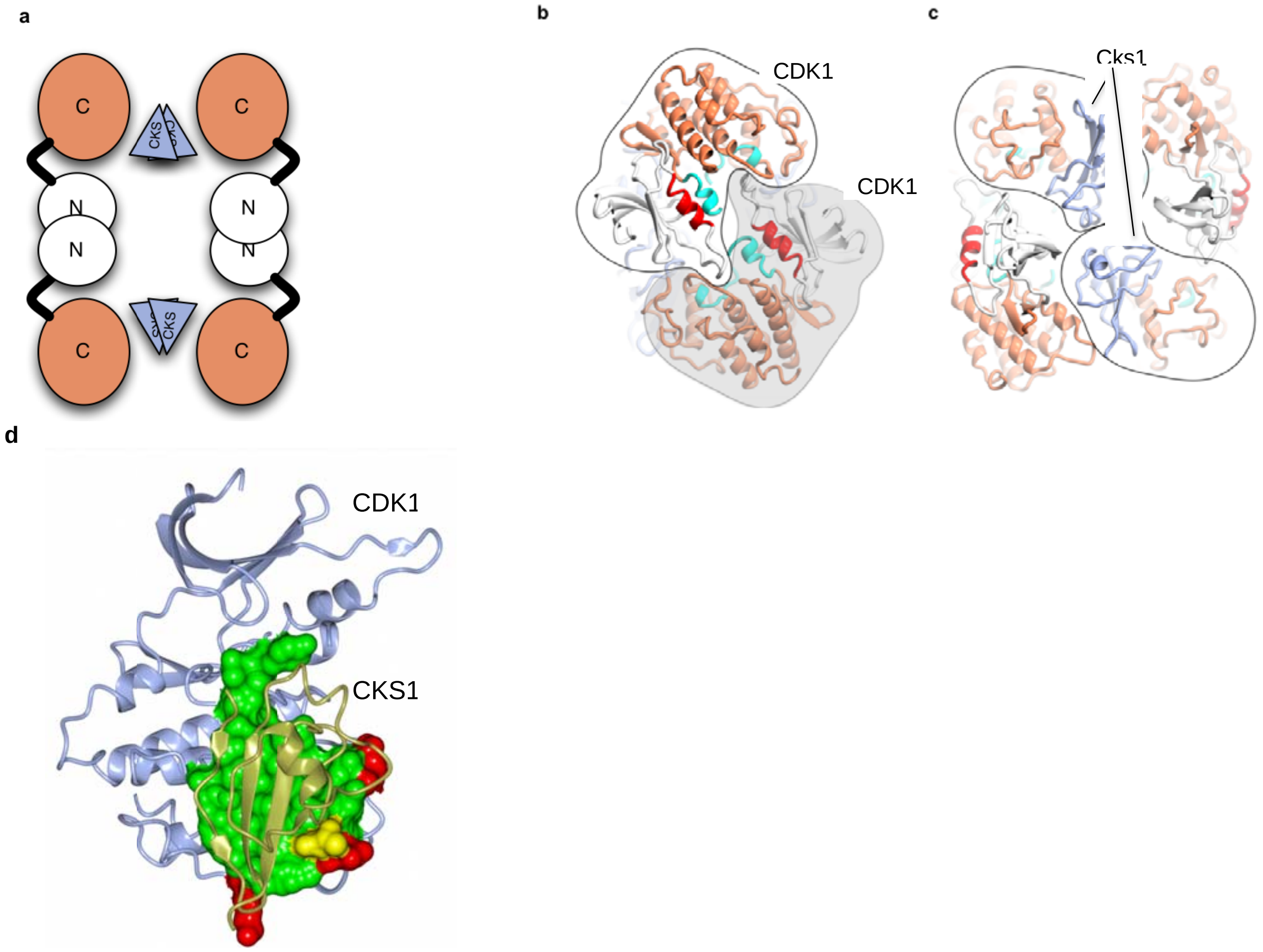

Supplementary Figure 1 | The CDK1-Cks1 crystal lattice. (**a**) Schematic of the CDK1-Cks1 crystal lattice. CDK1-Cks1 crystallizes in a lattice that contains 4 copies of the CDK1-Cks1 dimer in the crystallographic asymmetric unit. (**b, c**) CDK1-Cks1 packing within the crystal lattice. Each molecule is drawn in ribbon representation. Cks1 molecules are ice-blue and structural features of CDK1 are coloured as follows: N-terminal lobe, white; C-terminal lobe, coral; activation segment, cyan; C-helix, red. (**b**) The hairpin preceding αC in the CDK1 structure helps to stabilize the lattice as a pair of ncs-related CDK1 molecules exchange the hairpin so that it reaches across towards the reciprocal C-terminal lobe (chains C and E, and A and G respectively in PDB entry 4YC6). CDK1 molecules A and G are outlined. The tight packing of the lattice creates a second extended interface between each of these dimer pairs (**c**). The significant interaction across this interface effectively sandwiches each Cks1 molecule between two CDK1 C-terminal lobes. In this panel CDK1-Cks1 cognate pairs, A, B and E, F are outlined. (**d**) The CDK1-Cks1interface is highly conserved with the CDK2-Cks1 interface (PDB entry 1BUH). The CDK1 molecular surface is shown for residues within 7Å of Cks1, colored such that identical residues are green, conserved residues are yellow, while differing residues are red.

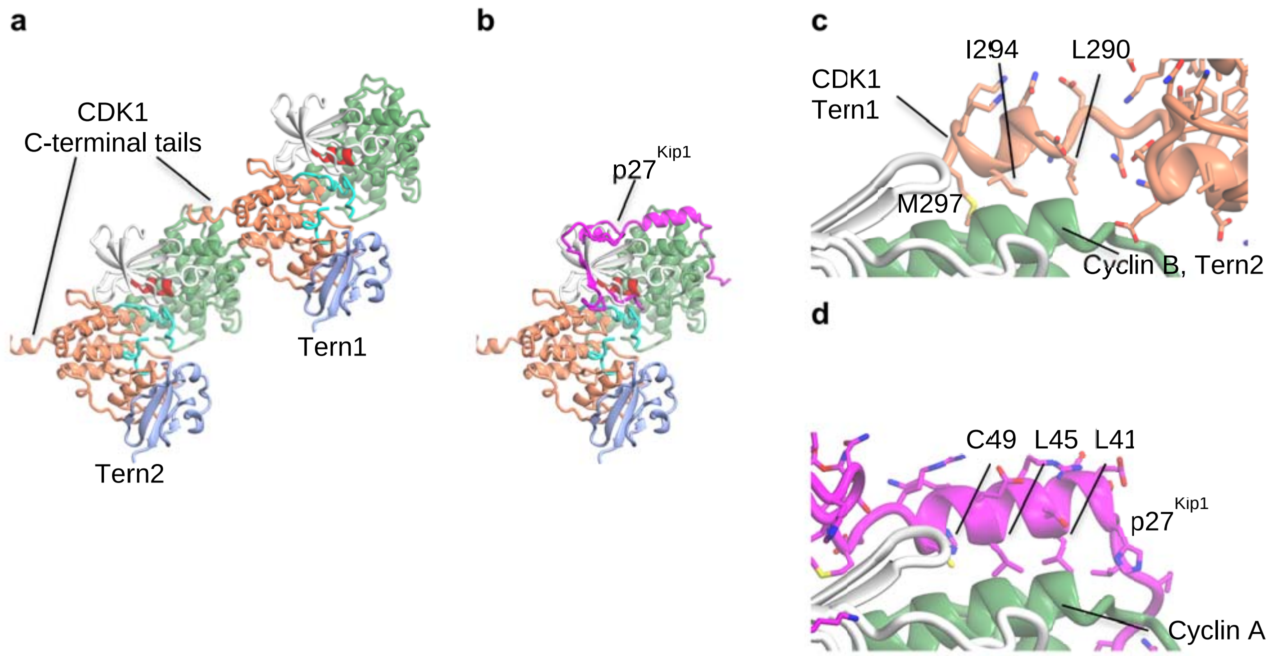

Supplementary Figure 2 | The CDK1 C-terminal helix is flexible and can bind to cyclin B. The C-terminus of CDK1 in apo CDK1-Cyclin B-Cks1 crystals forms a helix that mediates a lattice contact. (**a**) Two adjacent ternary complexes in the crystal. Ternary complexes are labeled "Tern1" and "Tern2" and are coloured according to the scheme used in Fig. 2. Cks1 molecules are ice-blue and structural features of CDK1 are coloured as follows: N-terminal lobe, white; C-terminal lobe, coral; activation segment, cyan; C-helix, red. The C-terminal residues of Tern1(284-297) form a helical excursion that sits in a groove on the surface of cyclin B in Tern2. (**b**) An equivalent groove on the surface of cyclin A forms part of the binding surface of $p27^{Kip1}$ in a ternary complex of CDK2-cyclin A-$p27^{Kip1}$. The structure of CDK2-cyclinA-$p27^{Kip1}$ (PDB code 1JSU) has been superimposed on ternary complex 2 (see panel (**a**)), by superimposing the corresponding cyclin subunits. The structure of Tern2 is drawn in ribbon representation, together with the structure of $p27^{Kip1}$, as extracted from the superimposed complex. (**c**, **d**) The C-terminal region of CDK1 forms an amphipathic helix that is capable of mimicking a part of $p27^{Kip1}$. Hydrophobic residues L290, I294, and M297 on one face of the helical C-terminal extension of CDK1 (**c**) recapitulate on a crystallographically-related cyclin B molecule the interactions made by residues L41, L45 and C49 of $p27^{Kip1}$ on cyclin A (**d**).

Cyclin B	169	170	173	174	177	253	257	258	260	265	266	283	286	287	294	295	297	299
	E	Y	D	I	Y	F	K	Y	E	E	I	R	E	M	N	F	L	R
Cyclin A	177	178	181	182	185	262	266	267	269	274	275	292	295	296	303	304	306	308
	D	Y	D	I	Y	L	K	F	E	E	V	L	E	H	T	F	L	A
Cyclin E	111	112	115	116	119	197	201	202	204	209	210	227	230	231	238	239	241	243
	A	N	E	V	I	F	K	L	E	K	L	L	E	L	K	W	L	P

Supplementary Figure 3| Analysis of the interfacial residues in the CDK1-cyclin B complex. Amino acids that mediate the CDK1-cyclin B interface were identified using the CCP4 program CONTACT [1]. Equivalent amino acids in cyclin A and cyclin E were identified by superimposing and inspecting the structures of CDK2-cyclin A (PDB code 1QMZ) and CDK2-cyclin E (PDB code 1W98) in CCP4MG.

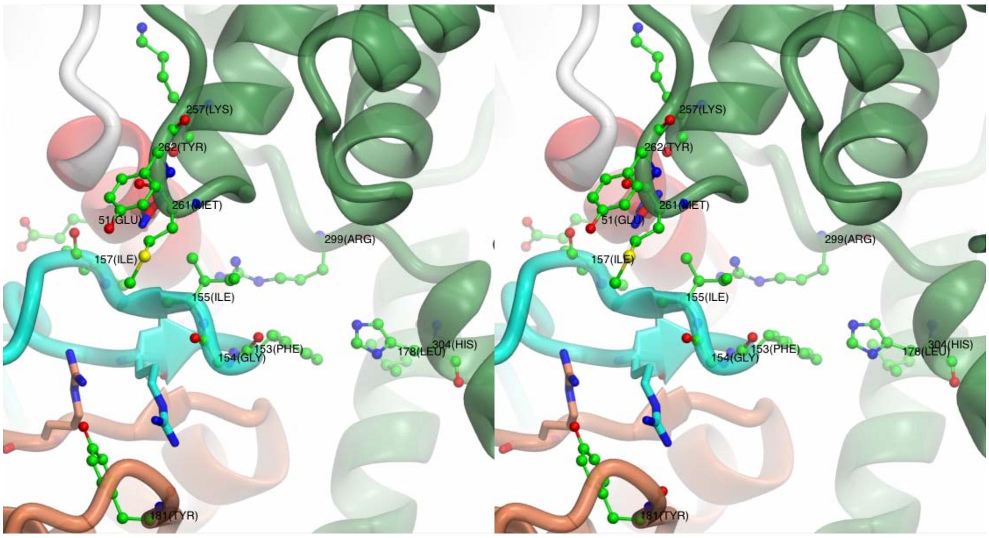

Supplementary Figure 4 | Structure of CDK1-cyclin B-Cks2. Stereo view with selected residues drawn in cylinder representation with carbon atoms coloured green. CDK2, cyclin B and Cks1 are coloured according to the scheme in Fig. 2. Cks1 molecules are ice-blue and structural features of CDK1 are coloured as follows: N-terminal lobe, white; C-terminal lobe, coral; activation segment, cyan; C-helix, red. Differences from the CDK2-cyclin A interface include the interactions of CDK1 residue F153, which sits within a cyclin B hydrophobic groove formed by the aliphatic portion of R299, L178 and H304. Differences in hydrophobic interactions are also observed. For example cyclin B residues M261 and Y262 at the end of helix α3 loop down to occlude CDK1155 from solvent, an interaction for which there is no equivalent in CDK2-cyclin A.

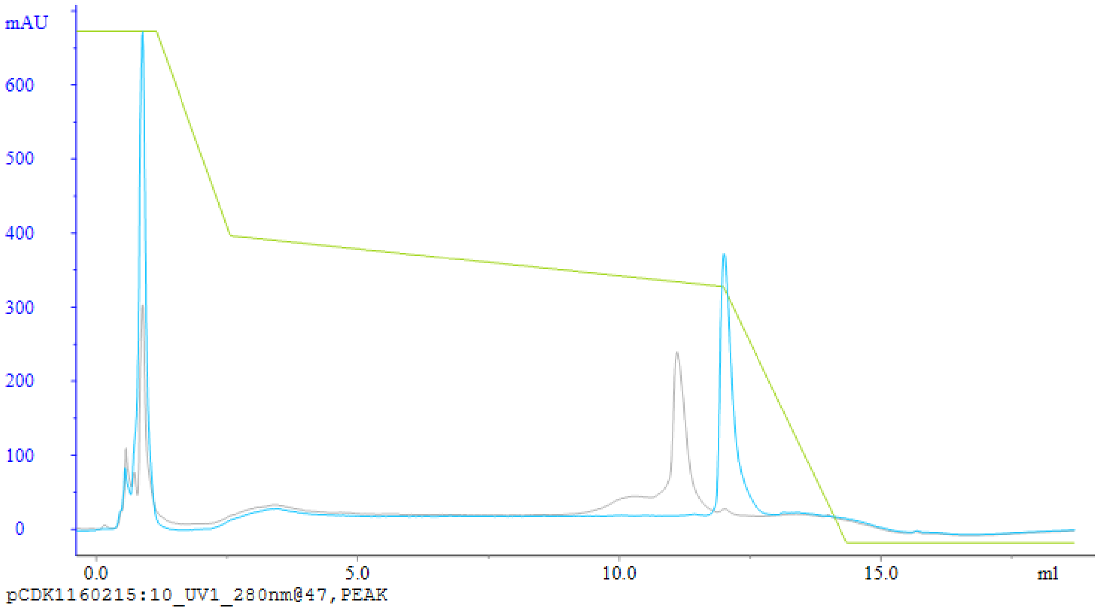

Supplementary Figure 5: High Performance Liquid Chromatography to assess the extent of CDK1 phosphorylation. Phosphorylated (grey) and non-phosphorylated CDK1 (cyan) can be resolved by reverse phase HPLC. A Jupiter 5u C4 300A column (Phenomenex) is equilibrated in 0.01% TFA in H_2O (buffer A) and then a 0.01% TFA/ acetonitrile (buffer B) gradient is developed from 50% to 60% Buffer B over 20 column volumes (represented by green line). Peaks of phosphorylated and unphosphorylated CDK1 are well resolved. The identity of the CDK1 species in each peak was confirmed by mass spectrometry.

a

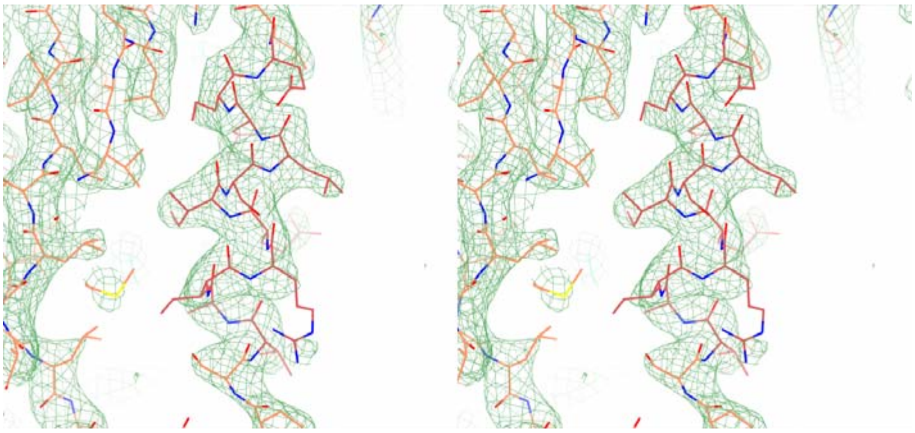

b

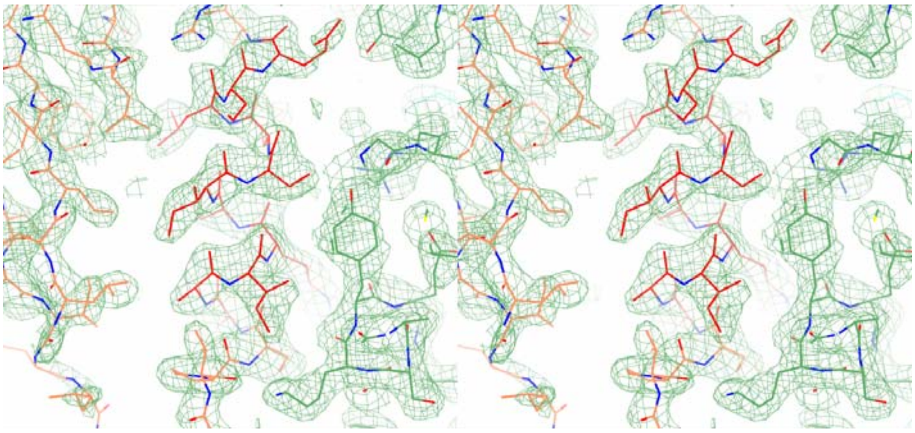

c

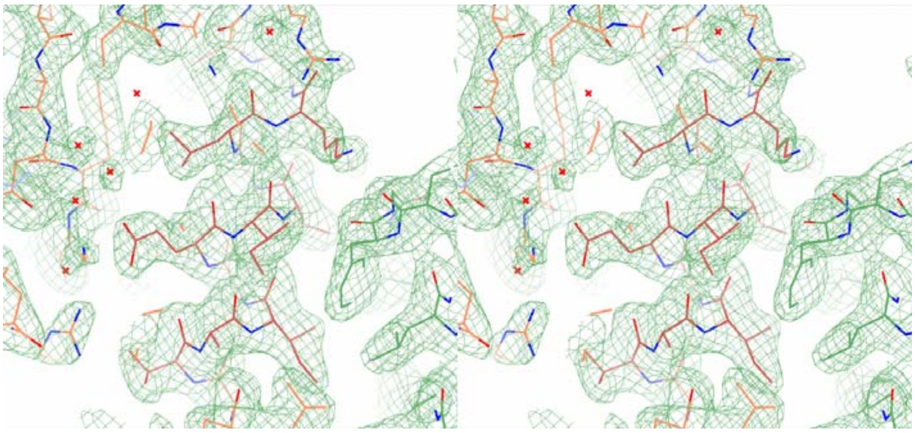

Supplementary Figure 6 | Representative electron density maps. Representative areas of the refined $2F_o$-F_c electron density maps for the structures of (**a**) CDK1-Cks1, (**b**) CDK1-cyclin B-Cks2 and (**c**) CDK1-cyclin B-Cks2-Compound 23. The maps are drawn as stereo pairs and contoured at 1σ respectively. The accompanying structures are drawn in ball and stick mode.

Supplementary Table 1| CDK substrate peptide sequences. The set of 8 peptides derived from the sequence of p107 starting at M618 vary systematically at the P+1 (proline or alanine), P+3 (serine or lysine) and recruitment site motif (KRXL or AAAA). In addition to these mutations the sequences contain the following mutations: Y648W, I674W and P651A (highlighted in italics). The peptides contain a GPLGS motif at the N-terminus which is a cloning artefact left after cleavage of the GST fusion with 3C protease. The residues from P to P+3 (where P is the site of phosphotransfer) are highlighted in bold and the residues at the recruitment site are underlined. Construct SPXS+RXL is the authentic p107 sequence at these motifs.

Peptide	Sequence
SPIS<br>+RXL	GPLGSMHPRVKEVRTDSGSLRRDMQPL**SPIS**VHER*W*SS*A*TAGSAKRRLFGEDPPKEMLMDK*W*
SPIK<br>+RXL	GPLGSMHPRVKEVRTDSGSLRRDMQPL**SPIK**VHER*W*SS*A*TAGSAKRRLFGEDPPKEMLMDK*W*
SPIS<br>-RXL	GPLGSMHPRVKEVRTDSGSLRRDMQPL**SPIS**VHER*W*SS*A*TAGSAAAAAFGEDPPKEMLMDK*W*
SPIK<br>-RXL	GPLGSMHPRVKEVRTDSGSLRRDMQPL**SPIK**VHER*W*SS*A*TAGSAAAAAFGEDPPKEMLMDK*W*
SAIS<br>+RXL	GPLGSMHPRVKEVRTDSGSLRRDMQPL**SAIS**VHER*W*SS*A*TAGSAKRRLFGEDPPKEMLMDK*W*
SAIK<br>+RXL	GPLGSMHPRVKEVRTDSGSLRRDMQPL**SAIK**VHER*W*SS*A*TAGSAKRRLFGEDPPKEMLMDK*W*
SPIS<br>-RXL	GPLGSMHPRVKEVRTDSGSLRRDMQPL**SPIS**VHER*W*SS*A*TAGSAAAAAFGEDPPKEMLMDK*W*
SAIK -<br>RXL	GPLGSMHPRVKEVRTDSGSLRRDMQPL**SAIK**VHER*W*SS*A*TAGSAAAAAFGEDPPKEMLMDK*W*

Supplementary References

1. CCP4. The CCP4 suite: programs for protein crystallography. *Acta Crystallogr D Biol Crystallogr* 50, 760-3 (1994).
